# GluK1 kainate receptors in parvalbumin interneurons modulate cortico-hippocampal network dynamics during social behavior

**DOI:** 10.1038/s41398-026-04060-z

**Published:** 2026-04-30

**Authors:** Jun Kyu Rhee, Simo Ojanen, Tiina Paakkunainen, Aino Vesikansa, Zoia Kharybina, Joni Haikonen, Rahaf Keskinen, Tomi Taira, Sari E. Lauri

**Affiliations:** 1 HiLIFE Neuroscience Center, University of Helsinki, Helsinki, Finland; 2 Department of Veterinary Biosciences, Faculty of Veterinary Medicine, University of Helsinki, Helsinki, Finland; 3 Molecular and Integrative Biosciences Research Program, Faculty of Biological and Environmental Sciences, University of Helsinki, Helsinki, Finland

**Keywords:** Learning and memory, Physiology, Neuroscience

## Abstract

The prefrontal cortex orchestrates complex behaviors by communicating with subcortical structures through synchronized oscillations. Here we show that ablation of GluK1 subunit-containing kainate receptors in parvalbumin interneurons (PV INs) disrupts oscillatory dynamics in the cortico-hippocampal circuits mediating social and cognitive behaviors. In control mice, the hippocampus-medial prefrontal cortex (HC-mPFC) circuit displayed elevated theta and gamma oscillation power as well as enhanced functional coupling during interaction with a familiar mouse. Similar circuit dynamics were not observed during interaction with a novel mouse, consistent with the idea that social recognition involves cortico-hippocampal communication. Mice lacking GluK1 in the PV INs (PV-*Grik1*^*-/-*^) showed defects in cognitive flexibility and social discrimination as well as impaired neurochemical phenotype of PV INs in the HC and mPFC. Electrophysiological recordings in the PV-*Grik1*^*-/-*^ mice revealed elevated theta and gamma oscillation power in both HC and mPFC along with enhanced functional coupling between these brain regions at rest. In contrast to the controls, no changes in the theta and gamma oscillation powers in the HC or mPFC or in the HC-mPFC coupling were detected in the PV-*Grik1*^*-/-*^ mice during social interaction. Our data suggest that impaired functional dynamics in cortico-hippocampal circuits in the PV-*Grik1*^*-/-*^ mice compromise social discrimination and shed light on the neurobiological mechanisms by which GluK1 dysfunction may contribute to neuropsychiatric disorders.

## Introduction

Parvalbumin-expressing GABAergic interneurons (PV INs) play a significant role in the pathophysiology of various neuropsychiatric disorders, as they are essential for generating the neuronal oscillations involved in cognitive functions [1–4]. PV INs exhibit low spike threshold, brief refractory period, and narrow spike waveform, qualities perfectly suited for rapid firing with minimal frequency adaptation [1, 3, 5]. In addition, PV interneurons responsible for perisomatic inhibition of pyramidal cells display gamma frequency (~30–80 Hz) resonance in response to stochastic excitatory conductance in vivo [6, 7]. These unique properties, together with their dense interconnectivity with pyramidal cells allow PV INs to efficiently modulate network oscillations in the gamma frequency [1, 8]. While essential for the generation of gamma oscillations, PV INs also modulate network activity at theta frequencies. Both phasic activation and phasic inhibition of PV interneurons trigger theta frequency resonance in pyramidal cells in vivo and in vitro [9, 10].

Precise coordination of network activity by PV INs depends on the glutamatergic excitatory inputs that recruit them [11–14]. Fast excitatory drive to PV INs is mediated by ionotropic glutamate receptors, which include NMDA receptors as well as calcium permeable AMPA receptors [15, 16]. AMPAR or NMDAR signaling deficiency in PV INs perturbs the oscillatory dynamics of neuronal networks and affects various cognitive behaviors [17–19]. PV INs also express GluK1 subunit-containing kainate-type glutamate receptors (GluK1 KARs), which have a critical role in development and maintenance of the PV IN phenotype [20, 21]. GluK1 KARs are broadly expressed in the developing brain and contribute to circuit maturation [22–27]; however, in the adult their expression is largely restricted to GABAergic interneurons [28]. Consistent with a critical role in regulating the physiological functions of PV INs, both GluK1 antagonism as well as targeted genetic ablation of the GluK1 gene, *Grik1* reduces the firing rate of PV INs in the amygdala and consequently, affects the balance between excitatory and inhibitory synaptic drive in the lateral amygdala microcircuit [20, 21]. Pharmacological and genetic manipulations of GluK1 KARs have been also implicated in regulation of theta and gamma oscillations in the hippocampus (HC), respectively [25, 29, 30]; however, the specific role of PV INs KARs in regulation of the circuit dynamics is not known.

This question is of importance, as genetic variants of GluK1 have been implicated in neuropsychiatric disorders such as schizophrenia, autism, and intellectual disability [31–33]. These disorders frequently manifest behavioral symptoms, including social deficits and cognitive inflexibility, which have been associated with the disrupted network dynamics and dysfunction of PV INs [34]. Specifically, PV IN dysfunction in the medial prefrontal cortex (mPFC) results in cognitive rigidity, which can be rescued by gamma frequency stimulation of PV INs in mouse models [34, 35]. During social behaviors, PV INs modulate the excitability and rhythmic activity of the principal neurons in both HC and mPFC, which is essential for the expression of social memory [36–40]. Anterograde and retrograde tracing studies have revealed that mPFC PV INs receive monosynaptic excitatory inputs from the hippocampal CA1 [41–43], providing a mechanism by which hippocampal output can efficiently orchestrate the temporal dynamics of the mPFC network contributing to social behaviors [38].

We set out to investigate how selective ablation of GluK1 KARs in PV INs alters the way corticolimbic network works in an awake behaving mouse when faced with increased demands on its memory and decision making. We demonstrate that impaired neurochemical phenotype of PV INs in the corticolimbic circuits and particularly in the mPFC of male mice lacking GluK1 in PV INs (PV-*Grik1*^*-/-*^) parallels defects in cognitive flexibility and social discrimination. Furthermore, we focus on characterizing the functional dynamics in the cortico-hippocampal circuitry during a social discrimination task, where the test mouse is allowed to freely interact with either a familiar (littermate) or a novel mouse. Our findings provide the first evidence implicating GluK1 KARs in regulation of the oscillation dynamics of cortico-hippocampal circuits responsible for social behavior in mice.

## Results

### Loss of GluK1 KARs results in low parvalbumin staining intensity within the cortico-hippocampal circuit

**Fig. 1:**
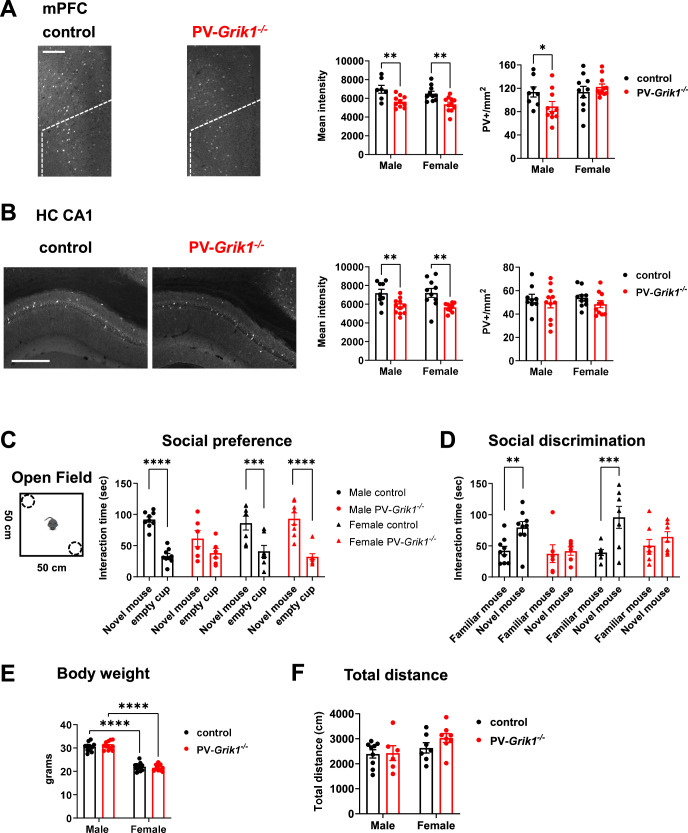
Perturbed neurochemical phenotype of PV interneurons in the cortico-hippocampal circuits in the absence of *Grik1* is associated with aberrant social behaviors. **A** Representative images of parvalbumin (PV) expression in the mPFC of the male control and PV-*Grik1*^*-/-*^ mice. Scale bar = 200 μm. Comparison of PV labeling intensity across genotypes and sexes. Effect of genotype, F_(1,34)_ = 20.59, p < 0.0001, 2-way ANOVA. **p < 0.01, Fisher’s least significant difference post hoc test. Comparison of PV+ cell density across genotypes and sexes. Effect of genotype and sex interaction, F_(1,34)_ = 4.031, p = 0.0527, 2-way ANOVA. *p < 0.05, Fisher’s least significant difference post hoc test. Males, control n = 7 sections(3 mice), PV-*Grik1*^*-/-*^ n = 10(3), Females, control n = 10(3), PV-*Grik1*^*-/-*^ n = 11(3). In all panels, bars represent mean ± SEM. **B** Representative images of PV expression in the HC CA1 of the male control and PV-*Grik1*^*-/-*^ mice. Scale bar = 500 μm. Comparison of PV intensity across genotypes and sexes. Effect of genotype, F_(1,36)_ = 18.96, p = 0.0001, 2-way ANOVA. **p < 0.01, Fisher’s least significant difference post hoc test. Comparison of PV+ cell density across genotypes and sexes. Effect of genotype, F_(1,36)_ = 1.724, p = 0.1975, 2-way ANOVA. Males, control n = 9(3), PV-*Grik1*^*-/-*^ n = 11(3), Females, control n = 10(3), PV-*Grik1*^*-/-*^ n = 10(3). **C** Top-down view of the 50 × 50 cm open field arena used for social preference and social recognition tests. The dashed circles indicate inverted transparent Plexiglas cups perforated with small holes. Analysis of the time spent interacting with a novel mouse vs empty cup, for male and female mice of both genotypes during 5 min test. Stimulus mice, genotype, and sex interaction, F_(1,52)_ = 5.068, p = 0.0286, ${\hat{\omega }}_{p}^{2}$ = 0.06, 3-way ANOVA. ***p < 0.001, ****p < 0.0001, Fisher’s least significant difference post hoc test. Males, control n = 9, PV-*Grik1*^*-/-*^ n = 6, Females, control n = 7, PV-*Grik1*^*-/-*^ n = 8. **D** Analysis of the time spent interacting with a novel vs familiar mouse, across genotypes and sexes during 5 min test. Stimulus mice and genotype interaction, F_(1,52)_ = 6.613, p = 0.0130, ${\hat{\omega }}_{p}^{2}$ = 0.08, 3-way ANOVA. **p < 0.01, ***p < 0.001, Fisher’s least significant difference post hoc test. Males, control n = 9, PV-*Grik1*^*-/-*^ n = 6, Females, control n = 7, PV-*Grik1*^*-/-*^ n = 8. **E** Body weights at P60-P70 across genotypes and sexes. Effect of genotype, F_(1,40)_ = 0.01317, p = 0.9092, effect of sex, F_(1,40)_ = 295.3, p < 0.0001, 2-way ANOVA. ****p < 0.0001, Fisher’s least significant difference post hoc test. Males, control n = 11, PV-*Grik1*^*-/-*^ n = 12, Females, control n = 11, PV-*Grik1*^*-/-*^ n = 10. **F** Total distance travelled during the habituation phase of the test across genotypes and sexes. Effect of genotype, F_(1,26)_ = 1.065, p = 0.3116, 2-way ANOVA. Males, control n = 9, PV-*Grik1*^*-/-*^ n = 6, Females, control n = 7, PV-*Grik1*^*-/-*^ n = 8.

In the amygdala, selective ablation of the GluK1 in the PV INs results in low density of PV expressing cells, characterized by diminished levels of PV expression and reduced excitability [21], marks of immature PV IN phenotype [44]. To verify whether loss of GluK1 KARs affect PV INs similarly in other corticolimbic brain areas, we analyzed PV and PNN expression in the HC and mPFC of the mice lacking *Grik1* expression in the PV INs (PV-*Grik1*^*-/-*^) and their littermate controls (*Grik1*^*tm1c*^; from here on, control) using immunohistochemistry. In both the HC and the mPFC, mean intensity of PV labeling in PV-*Grik1*^*-/-*^ mice of both sexes was lower than in littermate controls (Fig. 1). Notably, in mPFC of PV-*Grik1*^*-/-*^ males, also the density of PV positive cells was lower compared to controls (Fig. 1A), yet our data cannot distinguish whether this effect reflects loss of PV INs per se or decrease in PV expression level below the detection threshold. Intriguingly, in the mPFC, staining against perineuronal nets (PNN) revealed no significant differences between the genotypes, and the proportion of PV INs surrounded by PNNs was similar or even slightly increased in PV-*Grik1*^*-/-*^ mice (Fig. S1). Thus, GluK1 KARs affect the neurochemical phenotype of PV INs in the cortico-hippocampal circuits and particularly in the male mPFC, yet the effects of GluK1 ablation on PV expression in these brain regions were milder than what was previously observed in the amygdala [21].

### Social discrimination is perturbed in both male and female mice lacking GluK1 KARs in PV INs

To determine whether the absence of GluK1 KARs in PV INs affects social behaviors, we subjected PV-*Grik1*^−/−^ mice and their littermate controls to social preference and social discrimination tasks. Test mouse was placed in a 50 × 50 cm open field, containing two transparent Plexiglas cups, with age-matched familiar and unfamiliar mice. In the social preference task, one of the cups was empty. In contrast to controls, male PV-*Grik1*^*-/-*^ mice failed to display preference for the unfamiliar mouse over an empty cup, while female PV-*Grik1*^*-/-*^ mice displayed intact social preference (Fig. 1C). However, both male and female PV-*Grik1*^*-/-*^ mice failed to display preference for an unfamiliar mouse over a familiar mouse (Fig. 1D). While there is considerable variation in the data for both controls and PV-*Grik1*^*-/-*^ mice, the effect size (${\hat{\omega }}_{p}^{2}$) for the parameter interactions in the 3-way ANOVA were 0.06 and 0.08 respectively, indicating robust statistical significances with sufficient power.

The body weight of male and female PV-*Grik1*^*-/-*^ mice did not differ from controls (Fig. 1E); furthermore, the total distance travelled during the habituation phase of the test was not different between the control and the PV-*Grik1*^*-/-*^ mice, indicating that the observed social deficit is not due to PV-*Grik1*^*-/-*^ mice being less active (Fig. 1F). It should be noted, however, that in previous studies using a 30 × 30 cm open field test with brighter lights (~25 lux vs. ~150 lux), the total distance travelled was reported to be longer for both sexes of PV-*Grik1*^*-/-*^ mice than control mice, indicating that under more anxiogenic conditions, PV-*Grik1*^*-/-*^ can be hyperactive [21]. In the current setting, hyperactivity was not observed (Fig. 1F).

In order to separate between developmental and adult roles of GluK1 in PV INs, we used AAV viral vectors (mDlx-*Grik1*) to rescue GluK1 expression in the GABAergic INs in the mPFC and CA1 of male PV-*Grik1*^*-/-*^ mice. mDlx driven expression targeted primarily PV but also somatostatin (SOM) expressing INs (87 ± 8% vs. 13 ± 6% of infected neurons; respectively), the infection rate reaching 68 ± 6% of PV INs in the area CA1 (Fig. S2). PV and PNN staining intensities were significantly higher in mDlx-*Grik1* expressing PV INs compared to mDlx-EGFP expressing controls, further confirming the critical role of *Grik1* in regulating neurochemical phenotype of the PV INs [21; Fig. S2c). Behavioral testing indicated that the defects in social behavior were fully rescued by expression of mDlx-*Grik1* in the mPFC and HC of male PV-*Grik1*^*-/-*^ mice (Fig. S2d-f); mDlx-EGFP expression, used as a control, had no effect on the social behavior of female PV-*Grik1*^*-/-*^ mice.

Overall, these findings indicate that the absence of *Grik1* in PV INs is linked to significant deficits in social behavior, especially in the male mice.

### GluK1 KARs in PV INs regulate spatial relearning and cognitive flexibility in a sex-specific manner

**Fig. 2:**
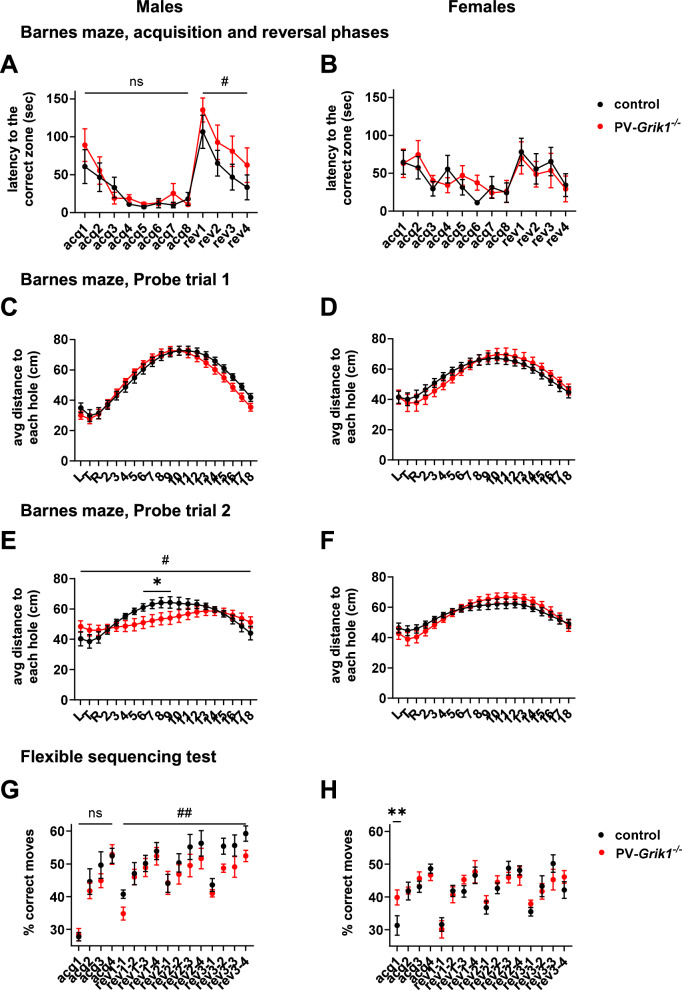
PV-*Grik1*^*-/-*^ male mice display impaired spatial relearning and cognitive inflexibility. **A** Comparison of trial-by-trial latencies to enter the correct zone with the escape box in the Barnes maze test, for littermate control and PV-*Grik1*^*-/-*^ males, during eight acquisition trials (acq1-8) and four reversal acquisition trials (rev1-4). Effect of genotype, F_(1,228)_ = 5.002, p = 0.0263, ${\hat{\omega }}_{p}^{2}$ = 0.015; during the acquisition phase, effect of genotype, F_(1,152)_ = 0.8201, p = 0.3666, ${\hat{\omega }}_{p}^{2}$ = 0.00, and during the reverse acquisition phase, effect of genotype, F_(1,76)_ = 4.697, ^#^p = 0.0334, ${\hat{\omega }}_{p}^{2}$ = 0.04, 2-way ANOVA. Control n = 10, PV-*Grik1*^*-/-*^ n = 11. In all panels, dots represent mean ± SEM. **B** Similar data as in A, for females. Effect of genotype, F_(1,228)_ = 0.01581, p = 0.9000, ${\hat{\omega }}_{p}^{2}$ = 0.00, 2-way ANOVA; during the acquisition phase, effect of genotype, F_(1,152)_ = 0.6240, p = 0.4308, ${\hat{\omega }}_{p}^{2}$ = 0.00, 2-way ANOVA, and during the reverse acquisition phase, effect of genotype, F_(1,76)_ = 0.3638, p = 0.4453, ${\hat{\omega }}_{p}^{2}$ = 0.00, 2-way ANOVA. Control n = 11, PV-*Grik1* ^*-/-*^ n = 10. **C** Comparison of average distance from each hole during the 1^st^ probe trial, for littermate control and PV-*Grik1*^*-/-*^ male mice. Effect of genotype, F_(1,380)_ = 2.896, p = 0.0896, ${\hat{\omega }}_{p}^{2}$ = 0.004, 2-way ANOVA. T is the hole where the escape box was during the acquisition phase. **D** Similar data as in C, for females. Effect of genotype, F_(1,380)_ = 0.0001, p = 0.9913, ${\hat{\omega }}_{p}^{2}$ = 0.00, 2-way ANOVA. **E** Comparison of average distance from each hole during the 2^nd^ probe trial, for littermate control and PV-*Grik1*^*-/-*^ male mice. Effect of genotype, F_(1,380)_ = 4.811, ^#^p = 0.0289, ${\hat{\omega }}_{p}^{2}$ = 0.01, effect of genotype and hole interaction, F_(19,380)_ = 1.882, ^#^p = 0.0143, ${\hat{\omega }}_{p}^{2}$ = 0.04, 2-way ANOVA. *p < 0.05, Fisher’s least significant difference post hoc test. T is the hole where the escape box was during the reverse acquisition phase. **F** Similar data as in E, for females. Effect of genotype, F_(1,380)_ = 0.2879, p = 0.5919, ${\hat{\omega }}_{p}^{2}$ = 0.00, 2-way ANOVA. **G** Intellicage flexible sequencing test. Comparison of trial-by-trial % correct moves made of male mice during both the acquisition and the three reversal phases. Effect of genotype, F_(1,176)_ = 7.594, p = 0.0065; during the acquisition phase, effect of genotype, F_(1,44)_ = 0.7388, ^ns^p = 0.3947, and during the three reversal phases, effect of genotype, F_(1,132)_ = 7.171, ^##^p = 0.0084, 2-way ANOVA. Reward chambers were swapped after acq4, rev1-4, and rev2-4. Control n = 6, PV-*Grik1*^*-/-*^ n = 7. **H** Similar data as in G, for females. Effect of genotype, F_(1,176)_ = 0.6898, p = 0.4074. **p < 0.01, Fisher’s least significant difference post hoc test; during the acquisition phase, effect of genotype, F_(1,44)_ = 2.612, p = 0.1132, and during the three reversal phases, F_(1,132)_ = 0.0090, p = 0.9244, 2-way ANOVA. Control n = 6, PV-*Grik1*^*-/-*^ n = 7.

We went on to investigate whether the sex differences observed in the effects of GluK1 KAR ablation on social behaviors also extend to spatial learning and relearning performance of the PV-*Grik1*^*-/-*^ mice. To this purpose, we used the Barnes maze test, where the mice are positioned in an aversive environment and are trained to find an escape box. Both male and female PV-*Grik1*^*-/-*^ mice learned the spatial location of the escape box in the Barnes maze equally well as the controls during the acquisition phase (Fig. 2A,B; for sex comparison of controls, see Fig. S3a). Also, no differences between the genotypes or sexes were detected during the 1^st^ probe trial assessing the spatial memory, i.e. ability to search for the hole where escape box used to be, the day after the conclusion of the acquisition phase (Fig. 2C,D). However, when the location of the escape box was changed in the reversal acquisition phase, the male PV-*Grik1*^*-/-*^ mice were significantly slower in relearning the new target location and performed worse in recalling the new location during the 2^nd^ probe trial compared to controls (Fig. 2A,E). In contrast, female PV-*Grik1*^*-/-*^ mice performed as well as the controls in relearning and recalling the new target location (Fig. 2B,F). The spatial relearning defects in male PV-*Grik1*^*-/-*^ mice were rescued by expression of mDlx-*Grik1* in mPFC and HC (Fig. S3), while mDlx-EGFP, used as a control, had no effect on spatial relearning performance in female PV-*Grik1*^*-/-*^ mice (Fig. S3).

To further assess the cognitive flexibility in the PV-*Grik1*^*-/-*^ mice, we employed a flexible sequencing task, where the mice learned to obtain a reward by sequentially shuttling between the two diagonally located chambers in the intellicage. After the training period, the locations of reward and non-reward chambers were swapped to test reversal learning; the reversal learning was repeated 3 times. Control and PV-*Grik1*^*-/-*^ mice of both sexes learned the rules equally well during training (Fig. 2G,H). However, while female PV-*Grik1*^*-/-*^ mice adapted to the rule changes, male PV-*Grik1*^*-/-*^ mice showed significantly lower number of correct visits than the controls when the locations of reward chambers were reversed (Fig. 2G,H). The average number of visits to the four test chambers was not different between the genotypes, indicating that the reduced adaptability to changing rules is not because male PV-*Grik1*^*-/-*^ mice were less motivated to visit the four test chambers (Genotype effect, males F_(1,11)_ = 1.206, p = 0.296; females F_(1,11)_ = 0.101, p = 0.757; 2-way ANOVA).

Thus, in the PV-*Grik1*^*-/-*^ mice, social and cognitive behaviors were more severely affected in males than females, a finding that aligns with the male-specific reduction of PV INs density in the mPFC.

### Head-fixed male PV-*Grik1*^-/-^ mice exhibit aberrant network activity in the hippocampus and in the mPFC at rest

**Fig. 3:**
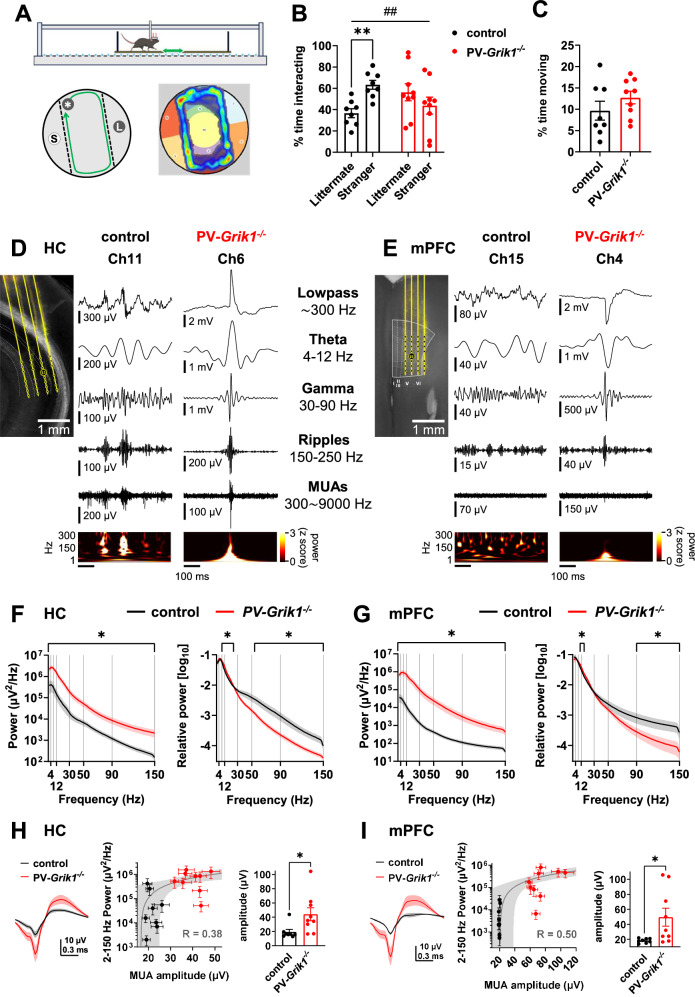
Head-fixed male PV-*Grik1*^*-/-*^ mice exhibit disrupted social behaviors and altered network activity both in the hippocampus and the mPFC. **A** Design of the experimental setup, where the test mouse is moving on a floating platform. A side view illustrates how the platform would move (green arrows) during locomotor activity of a head-fixed mouse. Bottom left, a top view. The dashed lines indicate flat welded wire mesh panels separating the littermate (L) and ICR novel stranger (S) from the test mouse (*). Bottom right, a heatmap showing the location tracking of a test mouse during a typical recording session. **B** Comparison of time spent interacting with either a littermate or a novel stranger over total social interaction time. Effect of genotype and stimulus mice interaction, F_(1,30)_ = 8.685, ^##^p = 0.0062, 2-way ANOVA. **p < 0.01, Fisher’s least significant difference post hoc test. Control n = 8, PV-*Grik1*^*-/-*^ n = 9. In all panels, bars, lines and shaded areas represent mean ± SEM. **C** Comparison of the time spent moving over total recording time. p = 0.256, unpaired t-test. Control n = 8, PV-*Grik1*^*-/-*^ n = 9. **D**,**E** Location of electrodes in the HC and mPFC and example traces of LFP from both genotypes with filtering to various frequency bands. Normalized 1–300 Hz LFP heatmaps are shown at the bottom. LFP traces are from an electrode located in the CA1 pyramidal cell layer and the mPFC layer 5. **F** Comparison of HC 2–150 Hz raw oscillation power at rest. *p < 0.05, Wilcoxon rank-sum statistic permutation test for multiple frequency bins. Comparison of HC 2–150 Hz relative oscillation power at rest. *p < 0.05, Wilcoxon rank-sum statistic permutation test for multiple frequency bins. Control n = 8, PV-*Grik1*^*-/-*^ n = 9. **G** Comparison of mPFC 2–150 Hz raw oscillation power at rest. *p < 0.05, Wilcoxon rank-sum statistic permutation test for multiple frequency bins. Comparison of mPFC 2–150 Hz relative oscillation power at rest. *p < 0.05, Wilcoxon rank-sum statistic permutation test for multiple frequency bins. Control n = 8, PV-*Grik1*^*-/-*^ n = 9. **H** Averaged multi-unit activity (MUA) waveforms and comparison of the mean MUA amplitudes between genotypes in the HC. **p = 0.0079, Mann-Whitney test. MUA amplitude and LFP power are significantly correlated, R^2^ = 0.38, linear regression; F_(1,143)_ = 14.33, p = 0.0002, Deming regression. **I** Similar data as in H, for mPFC. Genotype effect on MUA amplitude, *p = 0.0152, Mann-Whitney test. Correlation, R^2^ = 0.50, linear regression; F_(1,143)_ = 26.86, p < 0.0001, Deming regression. Control n = 8, PV-*Grik1*^*-/-*^ n = 9.

After confirming that genetic ablation of GluK1 in PV INs associates with cognitive behavioral defects, we went on to explore the physiological correlates of this phenotype using electrophysiological techniques. To this end, we first adapted the social recognition task for head-fixed mice, where the mouse is placed on a floating platform and is able to interact with a familiar littermate or a stranger mouse in separate compartments (Fig. 3A). Because the social deficit was more pronounced in the male PV-*Grik1*^*-/-*^ mice than the females, from here on, we focused on males only.

Analysis of the interaction times with a familiar littermate or a novel stranger indicated that the impaired social discrimination observed in the PV-*Grik1*^*-/-*^ mice is recapitulated in the head-fixed configuration. While the control mice spent more time interacting with a stranger than familiar littermate, similar difference was not observed in the PV-*Grik1*^*-/-*^ mice (Fig. 3B). The time spent moving during the test did not differ between the control and PV-*Grik1*^*-/-*^ mice (Fig. 3C). Furthermore, the number of littermate and stranger interactions were not different between the genotypes (F_(1,30)_ = 0.067, p = 0.79, 2-way ANOVA), indicating that outwardly quantifiable metrics of social interaction are not different between control and PV-*Grik1*^*-/-*^ mice. However, while the data is normally distributed without outliers (Table S5, S6), there is high variability in the time male PV-*Grik1*^*-/-*^ mice spent interacting with a stranger or a familiar littermate, which is not as obviously seen in the controls. It is possible that this reflects a phenotype of the PV-*Grik1*^*-/-*^ mice as high variability is also seen in social preference and social discrimination data of the freely moving mice (Fig. 1C,D).

After confirming that our head-fixed recording configuration is suitable for studying the network correlates of aberrant social discrimination in the PV-*Grik1*^*-/-*^ mice, we performed simultaneous recordings of local field potentials (LFP) and multi-unit activity (MUA) in the HC and the mPFC (Fig. 3D–I). Since ventral HC CA1 pyramidal cells project to the layer *5* pyramidal neurons in the PL of the mPFC [43, 45], channels located in layer *5* of the mPFC were selected for analysis. We first analyzed the baseline LFP powers in both brain regions at rest. The LFP power in all frequencies above 2 Hz in both HC and mPFC was higher in PV-*Grik1*^*-/-*^ mice compared to controls (Fig. 3F,G). Interestingly, however, normalizing the absolute power to the total power across all frequencies, the relative gamma power in both HC and mPFC was significantly lower in the PV-*Grik1*^*-/-*^ mice than the controls (Fig. 3F,G). Gamma power (30–90 Hz) was significantly higher in the HC than in the mPFC in control mice but not in the PV-*Grik1*^-/-^ mice (Fig. S4), indicating that brain region-dependent differences in the PV IN-dependent oscillatory activity were attenuated in the PV-*Grik1*^*-/-*^ mice.

The power spectra for both genotypes showed a clear peak in the theta frequency range, particularly in the HC. Antagonism of GluK1 subunit containing KARs has been previously shown to modulate the HC theta oscillations peak frequency [27]; likewise, we observed a significant difference in the theta oscillation peak frequency between the genotypes in the HC (control, 8.78 ± 0.11 Hz, PV-*Grik1*^*-/-*^, 9.46 ± 0.12 Hz; p = 0.0007, unpaired t-test) but not in the mPFC (control, 9.34 ± 0.23 Hz, PV-*Grik1*^*-/-*^, 9.64 ± 0.15 Hz; p = 0.264, unpaired t-test).

Analysis of MUA waveforms revealed a robust increase in the MUA amplitude in PV-*Grik1*^*-/-*^ mice compared to controls, both in the HC and in the mPFC (Fig. 3H,I). Individual recording’s average MUA amplitude was highly correlated to their corresponding 2–150 Hz LFP power (Fig. 3H,I). This result is consistent with a broadband increase in neuronal activity in PV-*Grik1*^*-/-*^ mice, which could be caused by network disinhibition resulting from impaired PV IN function.

These data confirm and expand the previous findings showing that reduced glutamatergic drive to PV INs results in enhanced oscillation power in the hippocampus as well as in the cortical networks [18, 19, 25, 46, 47], possibly because of a shift in the excitation/inhibition balance.

### Littermate interaction enhances theta and gamma power in the cortico-hippocampal circuit in control but not in PV-*Grik1*^-/-^ mice

To determine how the cortico-hippocampal networks are recruited during social behaviors, we next compared the HC and mPFC theta and gamma oscillation powers of both genotypes during movement and social interaction. Since the basal oscillation power was significantly different between the genotypes, we compared the fold increase in oscillation power over rest for each behavior.

**Fig. 4:**
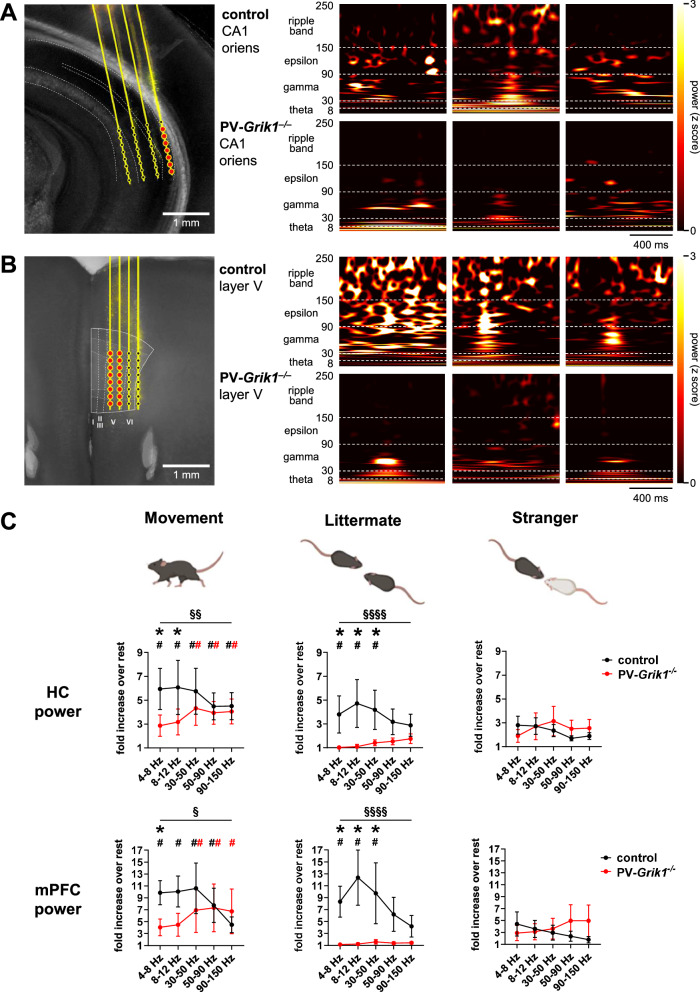
Selective ablation of GluK1 KAR in PV interneurons perturbs HC and mPFC theta and gamma oscillation dynamics during social interaction in male mice. **A**, **B** Location of electrodes in the HC CA1 oriens layer and the mPFC layer 5, and the normalized 1–250 Hz LFP power heatmaps of example epochs from three control and three PV-*Grik1*^*-/-*^ mice during littermate interaction. **C** Comparison of oscillation dynamics at various frequency bands during movement, littermate interaction, and stranger interaction. The data represent the mean LFP power fold increase ± SEM of a specific behavior from rest. For HC; movement, effect of genotype, F_(1,135)_ = 10.06, ^§§^p = 0.0019; littermate, effect of genotype, F_(1,135)_ = 26.41, ^§§§§^p < 0.0001; stranger, effect of genotype, F_(1,135)_ = 0.0432, p = 0.8356, 2-way ANOVA. *p < 0.05, Fisher’s least significant difference post hoc test. For mPFC; movement, effect of genotype, F_(1,135)_ = 4.711, ^§^p = 0.0317; littermate, effect of genotype, F_(1,135)_ = 37.77, ^§§§§^p < 0.0001; stranger, effect of genotype, F_(1,135)_ = 0.1876, p = 0.6656, 2-way ANOVA. *p < 0.05, Fisher’s least significant difference post hoc test. For each behavior and genotype, comparing the fold increase in LFP power of each frequency band to value 1. ^#^p < 0.05, one sample t-test against mean of 1. Control n = 8, PV-*Grik1*^*-/-*^ n = 9.

In the control mice, the HC and mPFC theta and low gamma (30–50 Hz) oscillation powers increased significantly during movement and littermate interaction, but not during stranger interaction (Fig. 4A–C). In PV-*Grik1*^*-/-*^ mice, the gamma oscillation power increased during movement in both HC and mPFC, while theta oscillation power was not significantly altered. Furthermore, neither theta nor gamma oscillation powers in PV-*Grik1*^*-/-*^ mice were affected during littermate and stranger interactions (Fig. 4C).

Thus, our data confirm the previous finding that social interaction enhances the theta and gamma oscillation powers in the mPFC [39, 48]; in addition, we show that interaction with a familiar littermate is associated with a significant increase in hippocampal theta and low gamma oscillation powers. Our data further suggest that absence of these oscillation dynamics in the PV-*Grik1*^*-/-*^ male mice correlates with the lack of social recognition memory exhibited during social discrimination tests.

### Social interaction associates with a shift in the hippocampal network state in control but not in PV-*Grik1*^-/-^ mice

**Fig. 5:**
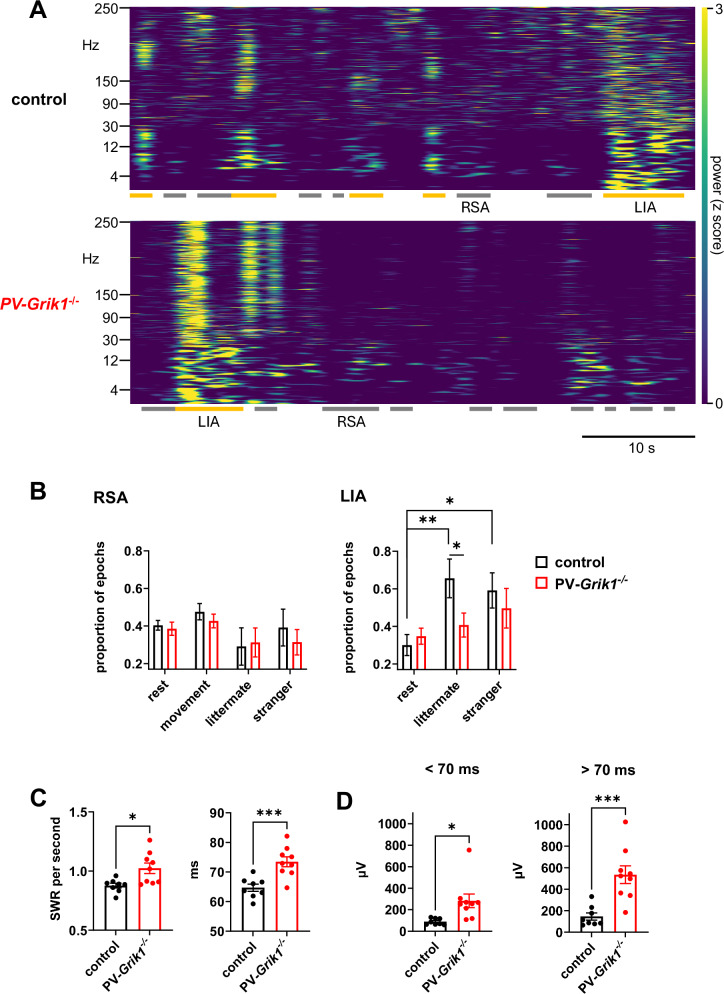
Hippocampal sharp wave ripples are altered in male PV-*Grik1*^*-/-*^ mice. **A** Normalized 0.3–250 Hz LFP power heatmaps of example HC recordings from control and PV-*Grik1*^*-/-*^ mice showing RSA in grey and LIA in orange. **B** Comparison of RSA prevalence across behaviors. Effect of genotype, F_(1,60)_ = 0.4501, p = 0.5049; Effect of behavior, F_(3,60)_ = 1.892, p = 0.1406, 2-way ANOVA. Comparison of LIA prevalence during rest, littermate interaction, and stranger interaction. Effect of genotype, F_(1,45)_ = 2.247, p = 0.1409; Effect of behavior, F_(2,45)_ = 4.681, p = 0.0142, 2-way ANOVA. *p < 0.05, **p = 0.0041, Fisher’s least significant difference post hoc test. Control n = 8, PV-*Grik1*^*-/-*^ n = 9. In all panels, bars represent mean ± SEM. **C** Comparison of average SWR frequency. *p = 0.0113, unpaired t-test. Comparison of average SWR duration. ***p = 0.0009, unpaired t-test. Control n = 8, PV-*Grik1*^*-/-*^ n = 9. **D** Comparison of average max amplitudes of SWRs shorter than 70 ms. *p = 0.0102, unpaired t-test. Comparison of average max amplitude of SWRs longer than 70 ms. ***p = 0.0008, unpaired t-test. Control n = 8, PV-*Grik1*^*-/-*^ n = 9.

During active behavior, HC exhibits specific activity patterns, also called network states, that correlate with specific behaviors and memory functions. Theta oscillatory activity or rhythmic slow activity (RSA) arises during tasks requiring voluntary movement, sensorimotor integration, and working memory (Fig. 5A) [49, 50]. Large irregular activity (LIA), marked by large amplitude oscillations over a broad range of frequencies (Fig. 5A) [51] during which trains of sharp waves often co-occur with 150–250 Hz high frequency ripples (SWR), is associated with non-locomotor and consummatory behaviors, such as immobility, eating, drinking, grooming, and are thought to promote memory consolidation and episodic memory retrieval [52, 53].

To characterize distinct states of activity during social interaction in control and PV-*Grik1*^*-/-*^ mice, we identified epochs of RSA and LIA in both genotypes and during different behaviors using a semi-automated method [51], where RSA and LIA were defined as periods with elevated theta/delta power ratio and total (2–80 Hz) power, respectively. Interestingly, although HC theta oscillation power increased during littermate interaction, we observed no difference in the occurrence of RSA during social interaction compared to rest in either genotype (Fig. 5B). However, LIA became more prevalent in the control mice during social interactions in a context invariant manner, i.e., larger proportion of epoch containing LIA were observed during both littermate and stranger interactions, compared to rest (Fig. 5B). Such change in the prevalence of LIA between rest and social interaction was not seen in PV-*Grik1*^*-/-*^ mice (Fig. 5B). Together with the lack of gamma and theta oscillation dynamics, these data support the idea that in the HC networks in PV-*Grik1*^*-/-*^ mice are unable to change their network states during social interaction.

We also looked at the occurrence and duration of SWRs during rest and social interaction. We found that SWRs were more frequent and longer in PV-*Grik1*^*-/-*^ mice than controls (Fig. 5C). This remained true when the duration of SWRs at rest and during social interactions were compared (rest p = 0.0009, social interaction p = 0.0012, unpaired t-tests; data not shown). In addition, in PV-*Grik1*^*-/-*^ mice, SWRs of similar durations showed larger amplitudes (Fig. 5D), pointing to recruitment of more or larger HC CA1 cell assemblies to generate SWRs in PV-*Grik1*^*-/-*^ mice.

Because ripples occurring in the mPFC are better characterized for non-REM sleep and quiet wakefulness [54] and granular/supragranular layers corresponding to layers II/III [55], which were not the focus of this study, we did not analyze them in any further detail.

### PV-*Grik1*^-/-^ mice exhibit aberrant functional coupling between HC and mPFC

**Fig. 6:**
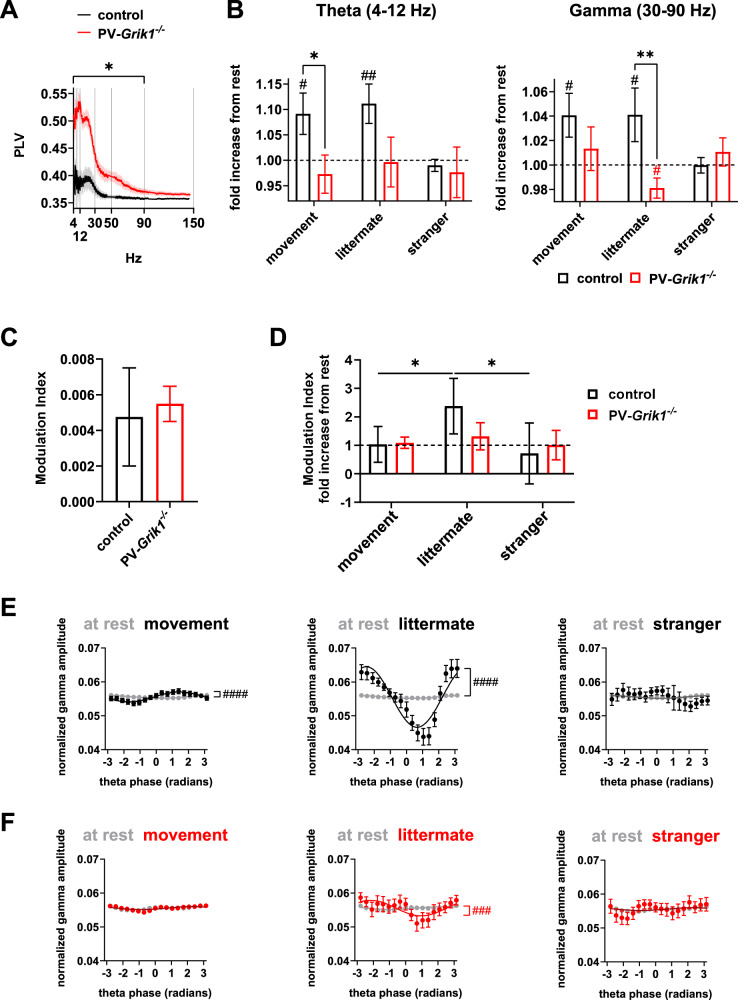
HC-mPFC oscillatory coupling at rest and during social behaviors is perturbed in male PV-*Grik1*^*-/-*^ mice. **A** Comparison of HC-mPFC 4–150 Hz phase locking values (PLV) at rest. *p < 0.05, Wilcoxon rank-sum statistic permutation test for multiple frequency bins. Control n = 8, PV-*Grik1*^*-/-*^ n = 9. In all panels, bars, dots, lines and shaded areas represent mean ± SEM. **B** Comparison of HC-mPFC 4–12 Hz theta and 30–90 Hz gamma coupling during movement, littermate interaction, and stranger interaction. The data represent PLV fold increase from rest. Theta, effect of genotype, F_(1,45)_ = 6.084, p = 0.0175; gamma, effect of genotype, F_(1,45)_ = 4.392, p = 0.0418, 2-way ANOVA. *p < 0.05, **p < 0.01, Fisher’s least significant difference post hoc test. For each genotype and behavior, comparing the fold increase in PLV to value 1. ^#^p < 0.05, ^##^p < 0.01, one sample Wilcoxon test against median of 1. Control n = 8, PV-*Grik1*^*-/-*^ n = 9. **C** Comparison of HC-mPFC theta gamma phase amplitude coupling (PAC) modulation index (MI) at rest. p = 0.2209, nested unpaired t-test. Control n = 8, PV-*Grik1*^*-/-*^ n = 9. **D** Comparison of HC-mPFC theta gamma PAC MI during movement, littermate interaction, and stranger interaction. The data represent MI fold increase from rest. Effect of genotype and behavior interaction, F_(5,35)_ = 2.602, p = 0.042, Mixed effects linear model REML. For control (n = 8), F_(2,15)_ = 3.858, p = 0.045, nested 1-way ANOVA, *p < 0.05, Fisher’s least significant difference post hoc test. For PV-*Grik1*^-/-^(n = 9), F_(2,20)_ = 0.517, p = 0.604, nested 1-way ANOVA. **E** Comparison of HC-mPFC 4–12 Hz theta oscillation phase nested 30–90 Hz gamma oscillation amplitudes in control mice. Rest vs movement (^####^p < 0.0001, F_(3,68772)_ = 19.11), rest vs littermate interaction (^####^p < 0.0001, F_(3,66234)_ = 97.20), and rest vs stranger interaction (p = 0.1184, F_(3,65892)_ = 1.955, sinusoidal curve fitting F test). Rest n = 3649 epochs(8 mice), movement n = 172(8), littermate interaction n = 31(8), stranger interaction n = 12(8). **F** Similar data for PV-*Grik1*^-/-^ mice. Rest vs movement (p = 0.44, F_(3,79554)_ = 0.9036), rest vs littermate interaction (^###^p = 0.0002, F_(3,73740)_ = 6.497), rest vs stranger interaction (p = 0.89, F_(3,73632)_ = 0.2085, sinusoidal curve fitting F test). Rest n = 4064(9), movement n = 356(9), littermate interaction n = 33(9), stranger interaction n = 27(9).

The altered theta and gamma dynamics in both the HC and mPFC of PV-*Grik1*^*-/-*^ mice suggested that cortico-hippocampal functional interactions may be affected in the mutants. Therefore, we next investigated the phase-phase coherence between HC and mPFC by calculating the phase locking value (PLV) across all frequency bands between HC CA1 and mPFC (PL layer *5*) LFPs in control and PV-*Grik1*^*-/-*^ mice. We found that the phase locking strength between HC and mPFC in both theta and gamma frequencies were significantly higher in PV-*Grik1*^*-/-*^ mice compared to controls at rest (Fig. 6A). In control mice, movement and littermate interaction but not stranger interaction, enhanced cortico-hippocampal PLV in both theta and gamma frequencies, whereas in PV-*Grik1*^*-/-*^ mice, similar increases were not observed (Fig. 6B). These data suggest that the elevated basal synchronization in PV-*Grik1*^*-/-*^ mice limits the inter-regional phase coding ability of the corticolimbic network during periods of demand on memory recall.

Inter-regional cross-frequency coupling within the corticolimbic network can also be quantified by phase-amplitude coupling (PAC) between HC theta and mPFC gamma oscillations. The modulation index (MI) [56], a measure of PAC between HC CA1 theta and mPFC PL layer *5* gamma oscillations during hippocampal RSA activity, was not different between PV-*Grik1*^*-/-*^ mice and controls at rest (Fig. 6C). In control mice, PAC increased during littermate, but not stranger interaction; however, similar effect was not observed in PV-*Grik1*^*-/-*^ mice (Fig. 6D-F). Thus, HC-mPFC theta-gamma PAC is a social context sensitive measure of cortico-hippocampal functional interaction, and loss of social context dependent modulation in PV-*Grik1*^*-/-*^ mice is correlated with their impaired social discrimination.

## Discussion

### Consequences of PV IN specific GluK1 ablation on PV phenotype and social behavior

Data from a variety of genetic models as well as patient samples suggest that altered synaptic inputs onto PV interneurons and concomitant disruption of oscillatory synchrony in the corticolimbic circuits are the proximal causes of social behavior deficits [8, 57–59]. PV interneuron specific NMDAR knockdown impairs short-term social memory ([60, 61, but see [18]), while reduced glutamatergic drive in PV interneurons of Erbb4 mutant mice correlates with pronounced disregard for the familiar mice in both the social preference and social discrimination tests [46]. Our previous work shows that genetic ablation of GluK1 KARs in PV INs results in significant reduction of glutamatergic drive to PV INs [21]. Accordingly, we find that loss of GluK1 KARs in PV interneurons results in significant defects in social behaviors with concomitant loss of PV expression throughout the corticolimbic network (for amygdala, see [21]).

Interestingly, even though the PV intensity was lower in the PV-*Grik1*^-/-^ mice, we detected no significant genotype differences in the PNN; if anything, the fraction of PVs with PNN was slightly higher than in controls in the PV-*Grik1*^-/-^ mice. On the other hand, both PV and PNN intensities were increased by mDlx-*Grik1* expression in adult PV-*Grik1*^-/-^ mice. Thus, *Grik1* expression regulates the neurochemical phenotype of PV INs [21], yet it appears that PV levels are more sensitive to changes in *Grik1* expression compared to PNN. Although the probability of the PV IN being surrounded by a PNN in general is highly dependent on its PV expression level, there is variability between brain regions [62]. This suggests that the PV-PNN association can be regulated, although the mechanisms underlying this regulation remain unknown.

PV IN specific ablation of GluK1 affected PV labeling intensity and social discrimination in both sexes. However, performance in social preference, spatial relearning, and cognitive flexibility tests was selectively impaired in males and correlated with decreased PV IN density in the mPFC, consistent with a central role of mPFC in these behaviors [34–36]. PV-*Grik1*^-/-^ mice display fear of novelty [21], which could contribute to these phenotypes. In addition, hormonal fluctuations modulate PV levels and PV IN excitability in females [63, 64] and we did not control the estrus cycle in our experiments; therefore, the sex differences observed in the present study should be interpreted with caution.

Behavioral characterization of mice lacking GluK1 has mainly focused on the anxiety-like behavioral phenotype that is detected in full knockdown model [65, 66] as well as conditional models where GluK1 expression is selectively silenced in GABAergic interneurons [25] or in the amygdala [20]. Cognitive rigidity has been previously observed in mice lacking GluK1 in GABAergic interneurons (GAD-*Grik1*^*-/-*^; [25]). Interestingly, GAD-*Grik1*^*-/-*^ males are hypoactive and show improved performance in the spatial relearning tasks, a phenotype that is opposite to that observed in PV-*Grik1*^*-/-*^ males. GluK1 kainate receptors are strongly expressed somatostatin (SOM) and cholecystokinin (CCK) expressing INs [67], which are targeted in GAD-*Grik1*^*-/-*^ but not in PV-*Grik1*^*-/-*^ mice. SOM and CCK interneurons have been implicated in learning behavior as well as in locomotion [68, 69]. Although the specific roles of *Grik1* in these INs are poorly understood, it is likely that the behavioral differences detected between GAD-*Grik1*^-/-^ and PV-*Grik1*^-/-^ mice depend on deficits in function of GABAergic INs that do not express PV. Understanding the behavioral significance of *Grik1* in SOM and CCK IN is an interesting target for future studies.

Mice lacking GluK2 KARs also display cognitive rigidity and social deficits [70], suggesting that these behavioral phenotypes may depend on heteromeric GluK1 and GluK2 subunit containing receptors in the PV INs. Since *Grik1* deficiency results in PV IN dysfunction already during development [21], these phenotypes could reflect developmental mis-maturation rather than adult kainate receptor function. However, both the PV levels as well as the social and spatial re-learning deficits in *PV-Grik1*^-/-^ mice were rescued by mDlx-*Grik1* expression in the adult HC and mPFC. Moreover, our previous findings show that local inactivation of GluK1 expression in adult amygdala resulted in PV IN malfunction, including low excitability, low glutamatergic synaptic inputs and low PV expression levels [21]. These data strongly suggest that expression of *Grik1* is needed to maintain PV INs functions in the adults.

### GluK1 KARs in PV INs regulate network activity in the HC and mPFC during social behavior

In several mouse models, loss of glutamatergic drive to PV IN manifests as elevated LFP power in corticolimbic circuits [18, 19, 25, 46]. These effects are to some extent postsynaptic receptor dependent; genetic ablation of NMDARs in PV INs results in an increase in HC gamma power [18, 19], while absence of GluA4 AMPARs in PV INs mainly affects SWRs [71]. Here, we find that ablation of GluK1 KARs in PV INs results in a broadband increase in the LFP power in both HC and mPFC as well as in increased occurrence of SWRs in the HC of awake behaving PV-*Grik1*^*-/-*^ mice at rest.

Our previous findings show that recruitment of PV INs in the amygdala of PV-*Grik1*^*-/-*^ mice is impaired due to the combined loss of synaptic GluK1 KAR signaling and immature PV IN firing properties [21]. We hypothesize that the consequent shift in E/I balance allows larger populations of principal neurons to participate in the oscillatory activity, observed as elevated MUA amplitude, basal LFP power, coherence, PAC, and larger and longer HC SWRs.

During social interaction, mPFC circuits synchronize to fire in low gamma and theta rhythms [38, 39, 48]. We found that in control mice, social interaction with a littermate led to a significant increase in low-gamma and theta power not only in the mPFC but also in the CA1 region of the HC, although the effect in the HC was less pronounced than that observed in the mPFC. In both brain areas, the dynamic increase in theta and low-gamma powers was not observed during stranger interaction, consistent with the hypothesis that ability to distinguish between familiar and unfamiliar conspecifics involves both HC and mPFC [38, 43, 72, 73].

In contrast to controls, no changes in the theta and gamma oscillation powers in either HC or mPFC were detected in PV-*Grik1*^*-/-*^ mice during littermate interaction. It is possible that the high basal power in the PV-*Grik1*^*-/-*^ mice resulted in limited ability to recruit and synchronize additional cell assemblies, particularly during theta activity. However, this explanation may be insufficient as significant changes in the LFP power in the PV-*Grik1*^*-/-*^ mice were observed during movement in both HC and mPFC. Therefore, the lack of oscillation dynamics in PV-*Grik1*^*-/-*^ mice could reflect a more specific inability to recruit PV INs in the HC and mPFC for retrieval of social information [37, 39]

### PV IN GluK1 ablation perturbs HC - mPFC functional coupling during social interactions

Theta oscillation coherence between HC and mPFC increases during tasks requiring integration of hippocampal spatial information into a broader decision making, including recognition memory retrieval for novelty discrimination [73, 74]. On the other hand, gamma oscillation coherence between HC and mPFC increases during successful cue encoding of spatial working memory [75, 76]. Accordingly, while hippocampal principal neurons in CA1 and CA2 subregions are critical in encoding and storage of social information [72, 77, 78], memory retrieval is thought to involve hippocampal communication with the mPFC. Functional coupling between HC and mPFC, driven by monosynaptic glutamatergic projections from pyramidal neurons of the ventral CA1 and subiculum to pyramidal neurons and GABAergic interneurons in the mPFC, and optogenetic inhibition of these projections disrupts novelty discrimination and social memory expression [38, 43, 45, 73].

Consistent with this scenario, we observed elevated HC-mPFC theta and gamma coupling as well as HC-mPFC theta-gamma PAC in control mice during littermate but not stranger interaction, a finding that is fully consistent with a role of the cross-frequency interactions in the HC-mPFC circuit in social discrimination.

In the PV-*Grik1*^*-/-*^ mice, strength of functional coupling between HC and mPFC was higher than in controls at rest. This phenotype is distinct from previously published mouse models, where PV IN dysfunction is associated with reduced functional coupling within the HC-mPFC circuit [46, 79], indicating that the long-range connectivity responsible for fast cortico-hippocampal synchronization is functional in the PV-*Grik1*^*-/-*^ mice. Interestingly, functional connectivity in corticolimbic circuits, assessed by fUS using BOLD signal, is slightly reduced in anesthetized PV-*Grik1*^*-/-*^ mice [21], indicating that some basal defects in the connectivity may exist. In contrast to controls, the HC-mPFC coupling was not enhanced during explorative activity or littermate interaction in the PV-*Grik1*^*-/-*^ mice. Furthermore, the strength of HC-mPFC theta-gamma PAC was responsive to social context in control mice, but not in PV-*Grik1*^*-/-*^ mice. Overall, these findings indicate that the impaired function of PV INs in PV-*Grik1*^*-/-*^ mice hampers the precise modulation of circuit dynamics essential for social behaviors. Together with impaired ability to adapt to changing rules in the Barnes maze and intellicage flexible sequencing task, these results point to general defects in accessibility and plasticity of neural ensembles encoding information about both social and non-social contexts.

It should be noted that all the recordings were done under head-fixed conditions, and hence sensory access constraints or stress caused by the head fixation might affect the results. The head-fixed configuration forces the test mouse to work with a conflicting vestibular input, resulting in vestibular-proprioceptive mismatch, which dampens HC theta power [80]. In addition, head-fixation could affect serum cortisol levels [81, 82]. Yet, these confounds are similar in both genotypes. Moreover, the defects in social discrimination in PV-*Grik1*^-/-^ mice were recapitulated under head-fixed configuration, suggesting that the observed differences in the mPFC-HC dynamics between genotypes are behaviorally relevant. However, we cannot exclude the possibility that under our recording conditions, some phenotypes may be masked.

In sum, we show that loss of GluK1 KARs in PV INs results in both HC-mPFC inter-regional and local HC and mPFC circuit dysfunction. This disruption in functional dynamics of the cortico-hippocampal network limits its capacity to meet the increased demands placed on both the local circuit and the inter-regional coupling during memory retrieval, and manifests as cognitive rigidity and diminished ability to distinguish between familiar and unfamiliar conspecifics.

## Materials and methods

Please see supplementary methods for more details.

### Animals

All experiments were performed using male and female mice lacking *Grik1* selectively in PV interneurons and their littermate controls. PV-*Grik1*^*tm1d*^ mice [21] heterozygous for PV-Cre were crossed with a conditional (floxed) line for GluK1 (*Grik1*^*tm1c/tm1c*^; [24]), to produce offspring with littermate controls (*Grik1*^*tm1c/tm1c*^) and mutants (PV-*Grik1*^*tm1d*^). All mice were of C57BL/6 J background. All the experiments were done in accordance with the University of Helsinki Animal Welfare Guidelines and approved by the Animal Experiment Board in Finland.

Mice underwent behavioral tests at P60-P70 and in vivo electrophysiology at P150-P180. Sex and age matched ICR mice were used as unfamiliar mice. The estrus cycle was not monitored.

### Immunohistochemistry

40 μm thick coronal sections of 4% PFA fixed P60-P70 brains were stained with anti-parvalbumin antibody (1:1000, PV235, SWANT), rabbit polyclonal anti-somatostatin antibody (1:1000, SAB4502861, Sigma-Aldrich), and biotin conjugated lectin from WFA (1:500, L1516, Sigma-Aldrich) as described [21].

### Viral constructs and injections

The cDNA encoding for rat Grik1-2b(Q) [83] and mDlx enhancer ([84]; Addgene plasmid #83900) were used to create mDlx-EGFP-P2a-GluK1-2b and mDlx-EGFP constructs. AAV8 viral particles were produced by Vector Biolabs. AAVs were bilaterally injected into the HC and mPFC of adult PV-*Grik1*^*-/-*^ mice: AP = 1.84 mm, ML = ± 0.28 mm, DV = −1.6 mm for mPFC, AP = −3.4 mm, ML = ± 3.3 mm, DV = −3.0/−2.3/−1.6 mm for HC, under deep isoflurane anesthesia as described [20].

### Behavioral tests

Social preference and social discrimination tests were carried out in a 50 × 50 cm open field arena with two transparent perforated Plexiglas cups. After habituation, an unfamiliar ICR mouse was placed randomly under one of the cups to test for social preference. In the second phase, another unfamiliar ICR mouse was placed under the remaining cup to test for social discrimination. The tests were recorded using EthoVision XT (Noldus, Wageningen, the Netherlands) under ~25 lux indirect lighting with supplementary infrared lighting. Barnes maze and intellicage flexible sequencing test were performed as described [25]. The investigator was blinded to the genotype during all tests.

### In vivo electrophysiology

Mice underwent head plate implantation surgery 3 weeks before the recording under deep isoflurane anaesthesia. Carprofen (Rimadyl vet, 5 mg/kg), dexamethasone (2 mg/kg), buprenorphine (Bupaq vet, 0.05 mg/kg), and lidocaine (0.5%) were used for pain management. Scalp was removed, the locations of craniotomies were marked (mPFC, AP = 2.165 mm, ML = 0.134 mm; HC, AP = −3.4 mm, ML = 2.65 mm), and a head plate and a grounding screw were attached to the skull.

After a week of recovery, the mice underwent gradual habituation to the experimental conditions and head-fixation (see supplementary methods). Littermates were allowed to interact with the test mouse during head-fixed habituation, but ICR mice remained strictly segregated.

Craniotomies were performed a day before the recording, as described [25]. For recordings, the mouse was head fixed on a floating platform and 32 channel silicone probes (NeuroNexus, Ann Arbor, MI, USA) were placed in mPFC and HC. Data was collected at 30 kHz using the SmartBox Pro (NeuroNexus, Ann Arbor, MI, USA), for 60 min. Probe positions were verified histologically. Mouse movements were tracked using both a video recording and a magnetic locomotion tracking system (Neurotar Oy, Helsinki, Finland).

### In vivo data analysis

All data were processed using custom Python scripts and publicly available packages (see supplementary methods for details).

Magnetic locomotion tracking system was used to annotate rests and movements. Video recording was used to annotate social interactions and groomings in 500 ms increments, and the two were time aligned and merged.

Channels for the prelimbic area layer *5*, hippocampal CA1 oriens, pyramidal, and radiatum layers were analyzed. Multi-unit activities were identified with Kilosort4 [85]. After automated adaptive common average referencing [86–90], down sampling and filtering, oscillation power was calculated using Morlet wavelet decomposition. Relative oscillation power was calculated by normalizing to the area under the raw oscillation power curve at rest. HC RSA and LIA epochs were identified as described with slight modifications [51, 91]. HC-mPFC PLV and theta-gamma PAC were calculated for all epochs and RSA epochs, respectively. HC SWRs were identified after 150–250 Hz filtering as described [92].

### Statistical analysis

Sample size was based on previous experience on similar experiments. Wilcoxon rank-sum statistic permutation tests for multiple frequency bins were computed using custom Python scripts. The rest of the statistics were computed using GraphPad Prism (Dotmatics, Boston, MA, USA) and custom R scripts.

## Supplementary information

Supplementary Methods

Supplementary Figures and Tables

## Data Availability

All data supporting the findings of this study are available within the paper and its Supplementary Information. Raw data are available from the corresponding author upon reasonable request.
