# Supplementary material for: GluK1 kainate receptors in parvalbumin interneurons modulate cortico-hippocampal network dynamics during social behavior
Source: Transl Psychiatry. 2026 Apr 30;16:313. doi: 10.1038/s41398-026-04060-z (PMC13276187; doi:10.1038/s41398-026-04060-z)
Supplement: Supplementary file 1 — Supplementary Methods [file 41398_2026_4060_MOESM1_ESM.pdf]

MATERIALS AND METHODS

Immunohistochemistry

P60-P70 mice were perfused with PBS and 4% PFA under ketamine-xylazine anesthesia. Immunostaining was carried out on 40 µm thick coronal sections as previously described (Haikonen et al., 2024), with mouse monoclonal anti-parvalbumin antibody (1:1000, PV235, SWANT, Burgdorf, Switzerland) and biotin conjugated lectin from WFA (1:500, L1516, Sigma-Aldrich, Darmstadt, Germany). For mDlx AAV injected mice, slices were also stained with rabbit polyclonal anti-somatostatin antibody (1:1000, SAB4502861, Sigma-Aldrich, Darmstadt, Germany).

Both medial prefrontal cortical (mPFC) and hippocampal (HC) sections were imaged with the AxioImager widefield light microscope with Apotome 2 (Zeiss, Oberkochen, Germany) using the identical light intensity and exposure time.

Images were analyzed using ImageJ (Schneider et al., 2012) and Zen microscopy software (Zeiss, Oberkochen, Germany) as follows:

For figure 1, for mPFC, cells in the prelimbic region and for HC, the CA1 region were analyzed. Regional regions of interest (ROIs) of similar size were chosen to quantify parvalbumin (PV) cell densities, perineuronal net (PNN) integrated optical densities, and PV PNN co-localization. ROIs were drawn around each PV+ cell to quantify the PV mean intensity. The data from individual cells was averaged for each section.

For figure S1, for mPFC, cells in the prelimbic and the anterior cingulate regions and for HC, the CA1 oriens layer and the adjacent superficial pyramidal layer were analyzed. Slices containing clearly visible PV+ cells were selected. ROIs were drawn around each PV+EGFP+ cell to quantify the PV and PNN mean intensities normalized to local background intensities. For mPFC, either the layer 1 or the area immediately surrounding the cells, and for HC, the area immediately surrounding the cells served as the background. To quantify the percentages of PV+ or SOM+ cells expressing AAV, the number of PV+EGFP+ or SOM+EGFP+ cells were divided by the total number of EGFP+ cells.

Viral constructs and injections

The cDNA encoding for rat Grik1-2b(Q) (Vesikansa et al., 2012) was subcloned into a pLen vector under mDlx enhancer (Dimidschstein et al., 2016; Addgene plasmid #83900). The mDlx-EGFP-P2a-GluK1-2b was transferred to AAV vector and AAV8-mDlx-EGFP-P2a-GluK1-2b and AAV8-mDlx-EGFP were produced by Vector Biolabs. AAVs were bilaterally injected into the HC and mPFC of adult PV-Grik1$^{-/-}$ mice under deep isoflurane anesthesia as previously described (Englund et al., 2021). Briefly, for mPFC, a single 100 nl injection was made at AP = 1.84 mm, ML = ±0.28 mm, DV = -1.6 mm, and for HC, three 100 nl injections were made at AP = -3.4 mm, ML = ±3.3 mm, DV = -3.0/-2.3/-1.6 mm, at the rate of 10-100 nl/min with 7-10 mins of wait time before withdrawing the injection needle.

Behavioral tests

Social preference and social discrimination

Social preference and social discrimination tests were done in three phases in the open field arena. First, the test mouse was released in the corner of a novel 50 X 50 cm open field arena with grey floors and white walls with two empty inverted transparent Plexiglas cups perforated with small holes in opposite corners and allowed to explore for 15 mins. Second, the mouse was removed from the arena, an unfamiliar sex and age matched ICR mouse was placed randomly under one of the transparent cups and the test mouse was returned to the arena for 15 mins. Third, the mouse was removed from the arena, another unfamiliar sex and age matched ICR mouse was placed under the remaining transparent cup and the test mouse was returned to the arena for 15 mins.

The experiment was performed under defuse indirect ~25 lux lighting and supplementary infrared lighting, and recorded and analyzed using EthoVision XT software (Noldus, Wageningen, the Netherlands).

Barnes maze

Barnes maze test was performed as described (Ojanen et al., 2023). The maze consists of a 100 cm diameter circular platform with twenty 5 cm diameter equidistant holes located around the perimeter (Ugo Basile, Gemonio, Italy). The escape box, a dark chamber filled with bedding material, was placed under one of the holes.

Two days before the start of the experiment, each test mouse was trained to enter the escape box and habituated for 10 seconds. For the acquisition phase, the test mouse underwent 2-3 acquisition trials per day: three on the 1st, 2nd day and two on the 3rd day, with at least 1 hour of rest between the trials. The mouse was given 180 seconds to find the escape box; each acquisition trial ended when the mouse either successfully entered the escape box or 180 seconds elapsed. If the mouse failed to find the escape box within 180 seconds, the mouse was gently guided to the box and allowed to enter the escape box on its own. On the 4th day, the day after the last acquisition trial, the test mouse underwent the first 90 second probe trial without the escape box. For the reverse acquisition phase, the test mouse underwent 2 reversal acquisition trials per day for two days immediately after the probe trial: two on the 4th and two on the 5th day. The escape box was placed under the hole on the opposite side of the Barnes maze for the reverse acquisition phase. After the last reversal acquisition trial on the 5th day, the test mouse underwent the second 90 second probe trial without the escape box.

The entire experiment was performed under the 500–600 lux bright lights to motivate the test mice to find and enter the escape box. EthoVision XT video tracking software (Noldus, Wageningen, the Netherlands) was used to record and analyze the data.

Intellicage

Flexible sequencing experiments were done in two phases in the IntelliCage (NewBehavior, TSE Systems, Berlin, Germany) apparatus designed to fit inside a large 610 X 435 X 215 mm cage (Tecniplast 2000P, Buguggiate, Italy) with bedding. The apparatus creates four test chambers in the four corners of the cage. The test mice entered and left each test chamber via 5 cm outer and 3 cm inner diameter tube outfitted with antennas to read the transponders as the mice traversed it. Each test chamber contained two 1.3 cm diameter openings (left and right) lined with photo beams designed to detect nose pokes attempting to access the bottle access-limited by motorized doors. Four red triangular shelters in the center of the IntelliCage are sleeping quarters that can double as stands to reach the food. RFID transponders (Datamars SA, Lamone, Switzerland) were subcutaneously implanted in all test mice for identification.

The mice were released in the center of the IntelliCage. For the first phase, the mice learned to obtain rewards (access to bottles) during specific hours using a single nose poke per visit to the test chamber. The mice first adapted to unlimited access to all bottles, and then the motorized doors to the bottles were closed and the mice learned to open the motorized doors and access the bottles for 5 secs with a single nose poke per visit to the test chamber. Finally, access to the bottles were limited to two 2-hour periods each day, e.g. between 4-6 and 20-22, and the mice learned to visit the test chambers only during those hours.

For the second phase, each test mouse was assigned a specific pair of diagonally located reward chambers where it could alternately access the bottles, i.e. a visit to one assigned chamber rendered that chamber “inactive” and the mouse could only access the bottle at the other diagonally located assigned chamber. The visits to the other two non-reward chambers were never rewarded. After 4 days, the assigned pair of reward chambers were swapped so the previously non-rewarding chambers were now rewarding, and the previously rewarding ones were no longer so. This reversal took place three times, each lasting 4 days, for a total of 16 days of experiment.

Visits to all test chambers were tracked over time using the Intellicage dedicated computer and software. An unbroken mouse movement starting from a test chamber and ending in a test chamber was quantified as a “move”. Moves were classified as “re-entry”, “diagonal”, or “adjacent”, and a diagonal move from one assigned chamber to the other assigned chamber was considered a correct move (Endo et al., 2011).

Design of the open field arena on a floating foam cage

325 mm diameter round foam cage bottoms with two magnets and 40 mm high circular plastic walls (Neurotar Oy, Helsinki, Finland) were outfitted with 2 flat welded wire mesh panels having a 6.4 mm mesh size and a 0.5 mm wire thickness (Schetelig Oy, Vantaa, Finland) to create an open field arena with 2 mouse-holding chambers with minimal nooks and crannies; the opening of the mesh was big enough for the mice to poke their noses through to explore the other side of the mesh, and the metal wire prevented the mice from chewing through and escaping. The wire mesh panels were made flush with the top of the foam cages, and the 2 compartments were covered with matching transparent red plastic films on top, creating a roof to encourage the mouse to stay in its chamber. Lightweight foam pieces (Tokmanni Oy, Mäntsälä, Finland) were placed in a few select spots where mice tended to hide to encourage it to explore rather than sit in a single spot in the open field. All the components were held together by tape for easy disassembly, cleaning, and reassembly. The total weight of the open field arena on a floating foam cage came out to be about 70-80 grams. In total, the experiment required 12 open field arenas on floating foam cages.

In vivo electrophysiology

Head plate implantation surgery

All mice were single housed 1 day prior to the head plate implantation surgery. On the day of the surgery, the mice were first weighed and then anesthetized with ≥ 4% isoflurane in an induction chamber, moved to a 37 °C heating pad in the stereotaxic frame, and then maintained on 1.5-2 % isoflurane for the duration of the surgery. Both eyes were covered with ophthalmic ointment (Viscotears, Novartis), and carprofen (Rimadyl vet, 5 mg/kg), dexamethasone (2 mg/kg) and buprenorphine (Bupaq vet, 0.05 mg/kg) were administered subcutaneously. The hair on top of the head was trimmed and the skin was disinfected with povidone-iodine (Betadine). Lidocaine (0.5%, max 5 mg/kg) was injected under the scalp for local analgesia and then the scalp and periosteum were removed. The surface of the skull was roughened using a large diameter drill bit and carefully cleaned. The edges of the wound were sealed with the tissue adhesive (Vetbond, 3M). The locations of craniotomies were identified and inked in skull with a needle (AP = 2.165 mm, ML = 0.134 mm for mPFC, and AP = -3.4 mm, ML = 2.65 mm for HC). A head plate (model 1, Neurotar, Helsinki, Finland) weighing 1 gram was attached to the skull using a small amount of superglue (Loctite precision) so that the mark for mPFC was inside the head plate and the mark for HC was outside of the head plate with enough clearance around the marks. A grounding screw was attached to the skull on the opposite side using dental cement (RelyX, 3M). The sides of the head plate and all visible areas of the skull were covered with the dental cement and cured with UV light. The mice were given intraperitoneal injections of saline during the surgery to prevent excessive dehydration. The mice were allowed to recover on the heating pad in their home cages and provided with water-soaked soft food pellets within easy reach. The mouse was monitored for any weight changes and was given carprofen (Rimadyl vet, 5 mg/kg), and buprenorphine (Bupaq vet, 0.05 mg/kg) intraperitoneally for three days following the surgery. The mice were allowed to rest and recover in the home cage for at least a week before starting the habituation protocol. If the mice lost more weight than allowed in the approved animal experiment protocol, the mice were euthanized; however, if the mice simply did not gain enough body weight in a week for the strength necessary for the habituation phase, they were allowed to rest another week.

Habituation

After recovering from the implantation surgery for a full week, the mouse in its home cage was first moved to the experimental room with a working Neurotar Mobile HomeCage® Large air dispenser for 60 mins once a day for 2 days of passive environmental habituation. The mice were then handled by the experimenter for 7 mins twice a day for 3 days of experimenter habituation. Lastly, the mice underwent head-fixed habituation in the open field arena on a floating foam cage for 5, 10, 15, 30, 60, 60 mins once a day for 6 consecutive days. To (1) develop the strength necessary to maneuver the floating foam cage as it will be on the recording day, and (2) give the test mouse ample opportunity to

get reacquainted with its littermate from whom it had been separated since the day of the surgery, we placed a littermate in one chamber and 40 gram metal weight in the other chamber throughout the head-fixed habituation phase. The littermates standing in as familiar mice underwent identical passive environmental and experimenter habituation until the open field arena habituation when it was allowed to interact with the test mouse.

For the entire duration of the habituation, the 4 ICR mice standing in as novel mice were always temporally and spatially segregated from the test mouse. After undergoing identical passive environmental and experimenter habituation, the 4 ICR mice were habituated in the open field arena on a floating foam cage in one of the two chambers, with the other chamber holding a 40-gram metal weight. The experimenter arbitrarily moved the floating foam cage with fingers to mimic the floating foam cage being moved by the head-fixed test mouse on the recording day. The ICR mice were given about a week's break before going through identical habituation as its next paired experimenter mouse.

After each head-fixed habituation session, the open field arena on the floating foam cage was completely disassembled and all parts including the foam cage thoroughly washed in unscented detergent to minimize traces of the previous habituation.

Craniotomy and electrophysiology

The day after the last 60 mins head-fixed habituation, a craniotomy was performed over right medial prefrontal cortex and right posterior hippocampus under anaesthesia, as described (Ojanen et al., 2023). The craniotomies were covered with silicone adhesive (Kwik-Sil, World Precision Instruments, Sarasota, FL, USA), and a bath was constructed on top of the opening of the head plate by making approximately 3-5 mm high walls out of Kwik-Sil. The mice were allowed to recover on the heating pad in their home cages and provided with water-soaked soft food pellets.

The recording took place after a full day of recovery. The mouse was head fixed on the floating platform and after removing the Kwik-Sil plugs covering the craniotomies and attaching the ground wire to the grounding screw, two 32 channel silicone probes (A4x8-5mm-177-50-200 and A4x8-5mm-177-100-200, NeuroNexus, Ann Arbor, MI, USA) stained with DiI dye (V22885, ThermoFisher, Waltham, MA, USA) were sequentially placed over the craniotomies and lowered. First the four shanks of A4x8-5mm-177-100-200 probe were aligned and angled 15 degrees in the coronal plane and lowered to the depths of 2.83 mm to target the HC. Then the four shanks of a A4x8-5mm-177-50-200 probe were angled 45 degrees in the transverse plane (the most medial shank is the most rostral, and the most lateral shank is the most caudal) and lowered to the depths of 2.16 mm to target the mPFC.

The baths surrounding the craniotomies were filled with 0.9 % sterile filtered saline, and after 5 minutes of stabilization, started recording at 30 kHz using the SmartBox Pro (NeuroNexus, Ann Arbor, MI, USA). After the recording, the craniotomies were covered with Kwik-Sil and the mouse was returned to the home cage. The mice were perfused with PBS followed by 4% PFA and the brains were collected for histological verification of probe positions.

The location of the head-fixed mouse on the floating foam cage was tracked with a video camera (Microsoft LifeCam Cinema, Microsoft, Redmond, AW, USA) at 1280 x 720 pixels, 30 frames per second, and a magnetic locomotion tracking system following the position of two magnets attached to the bottom of the foam cage (Neurotar Oy, Helsinki, Finland) at 250 Hz.

<u>In vivo data analysis</u>

All in vivo electrophysiology experiment related data were processed using custom Python (v 3.11.4) scripts written with MNE (v 1.4.2, Gramfort et al., 2013), MNE-connectivity (v 0.5.0), Scipy (v 1.11.1, Virtanen et al., 2020), Scikit-learn (v 1.3.0, Pedregosa et al., 2011), Scikit-image (v 0.21.0, van der Walt et al., 2014), Ripple-detection (v 1.5.1, Kay et al., 2016) packages.

Identification of rest, movement, social interactions, and grooming epochs

When the speed of mouse detected by magnetic locomotion tracking system exceeded 8 mm/sec the mouse was classified as *moving*; otherwise, the mouse was classified as *resting*. Periods of *social interactions* and *grooming* captured by the video camera were manually annotated in 500 ms

increments and combined with move/rest annotations extracted from the magnetic locomotion tracking system by synchronizing the hardware and software time stamps using a custom Python script. Each recording's combined annotation was manually verified at two randomly sampled time points to ensure that the timeline of events is consistent between the combined annotation and both the magnetic locomotion tracking system and the video camera footage. Because the median duration of social interactions was 1 second for both control and PV-*Grik1*$^{-/-}$ male mice, all behaviors were subdivided into 1 second epochs. When a behavior period did not neatly subdivide into 1 second epochs, the epochs were spread out evenly within the period to ensure even coverage.

Analysis of oscillation power, coherence, theta-gamma phase amplitude coupling, and hippocampal sharp wave ripples (SWR), and multi-unit activities (MUA)

Channels for the prelimbic area layer 5, hippocampal CA1 oriens, pyramidal, and radiatum layers were chosen based on DiI dye traces of the probes in fixed brains.

For oscillation power analysis, the data was then processed via automated adaptive common average referencing (Ludwig et al., 2009; Kelly et al., 2013; Islam et al., 2014; Xinyu et al., 2017; Khorasani et al., 2019), down sampled to 1 kHz, low pass filtered at 300 Hz, and notch filtered at multiples of 50 Hz. Time frequency decomposition using Morlet wavelet was performed over 2-150 Hz in steps of 0.1 Hz (for 2-12 Hz), 0.5 Hz (for 12-90 Hz), 1 Hz (for 90-150 Hz). Raw oscillation powers between male control and PV-*Grik1*$^{-/-}$ mice were first tested to see if the oscillation power difference in each frequency bin was significantly less likely than the chance differences generated by 1000 randomly permutated samples of equal sizes (Maris et al., 2007). Once we confirmed that there were significant differences across all frequencies, to compare the behavior oscillation power changes between male control and PV-*Grik1*$^{-/-}$ mice, we divided the raw behavior oscillation powers by average total power or area under the curve of the raw rest oscillation power to derive normalized behavior oscillations powers. The normalized oscillation powers were averaged in following frequency bins for further comparison: low theta (4-8 Hz), high theta (8-12 Hz), low gamma (30-50 Hz), high gamma (50-90 Hz), and epsilon (90-150 Hz).

For phase locking analysis, the data was down sampled to 1 kHz, low pass filtered at 300 Hz, and notch filtered at multiples of 50 Hz. Phase locking values (PLV) of the theta (4-12 Hz) and gamma (30-90 Hz) oscillations were computed and averaged between each channel in the prelimbic area layer 5 and the hippocampal CA1 using *spectral_connectivity_time* function of the MNE-connectivity package (Vinck et al., 2011; Yoshinaga et al., 2020).

For theta-gamma phase amplitude coupling, the data was down sampled to 1 kHz, low pass filtered at 300 Hz, and notch filtered at multiples of 50 Hz. The normalized amplitudes of the low gamma (38-43 Hz) oscillations per 1/18 radians of theta (7.7-9.5 Hz) phase and the modulation indices (Hülsemann et al., 2019) between the prelimbic area layer 5 channels and hippocampal CA1 channels identified as having rhythmic slow activity (RSA) were calculated. For the normalized theta phase nested gamma amplitude plots, if the sum-of-squares F test for least squares regression fitting of the sinusoidal curves was significant, separate curves were fitted.

For detecting hippocampal SWR, *Kay_ripple_detector* function of the ripple-detection package (Kay et al., 2016) was used. Hippocampal CA1 pyramidal cell layer data was down sampled to 1 kHz and band pass filtered at 150-250 Hz. Because PV-Grik1$^{-/-}$ display larger baseline amplitude power fluctuations, to prevent inappropriate identification of spurious activities as SWRs, only the putative SWRs whose amplitude exceeded 2 standard deviations of the epoch baseline power fluctuation for at least 15 ms were chosen as SWRs. ~1 % of the identified SWRs were visually verified.

For identifying MUAs, the data was processed using Kilosort4 (Pachitariu et al., 2024) without drift correction or allowing for templates from the data, and with the following adjustments: scale = 1000 to set amplitudes to uV, dminx = 250 to equal the spacing between the shanks, and max_peels = 5 to reduce the iterations during the matching pursuit step that won't help in isolating single units.

Identification of rhythmic slow activity (RSA) and large irregular activity (LIA) epochs

RSA epochs were identified as epochs where the power in the 6-12 Hz frequency band divided by the power in the delta (2-4 Hz) frequency bands was 1 standard deviations above the mean for longer than 1 second (Csicsvari et al., 1999; Malezieux et al., 2020). LIA epochs were identified as epochs where power in the 2-80 Hz frequency band was 0.25 standard deviations above the mean for longer than 1 second when the mouse was not moving (Malezieux et al., 2020). For calculating the prevalence of RSA and LIA epochs, epochs where any channel in the region of interest displayed RSA activity were considered RSA epochs and similarly if any channel displayed LIA activity, the epoch was considered an LIA epoch.

Statistical analysis

Wilcoxon rank-sum statistic permutation tests for multiple frequency bins were computed using custom Python scripts. First, the distribution of control and PV-*Grik1*$^{-/-}$ datasets were each checked for normality using Anderson-Darling, Shapiro-Wilk, and Kolmogorov-Smirnov tests. If the datasets were robustly normally distributed, t-test was used, and if not, Wilcoxon rank-sum statistic was used to compute each frequency for significance $p<0.05$, and significant contiguous frequency clusters were identified. The datasets were then combined and randomly shuffled and the test repeated for 1000 permutations. Using the 95 percentile of the significant frequency cluster size distribution as the significant cluster size threshold, the significant contiguous frequency clusters from the original control and PV-*Grik1*$^{-/-}$ comparisons were identified.

The rest of the statistics were computed using GraphPad Prism (v 10.4.1, Dotmatics, Boston, MA, USA) and custom R scripts.

For standalone determination of normality for values and their residuals, D'Agostino-Pearson, Anderson-Darling, Shapiro-Wilk, and Kolmogorov-Smirnov normality tests in GraphPad Prism were employed. For all stand-alone pairwise comparisons, unpaired two tailed t-tests were computed using GraphPad Prism.

For 3way ANOVA and 2way ANOVA, either (1) the data were fit to full models including the interaction effects, or to models with only main effects for Mixed effects linear model using GraphPad Prism, or (2) the data were fit to linear models with explicit interaction terms using a R script.

For (1) 3way ANOVA and Mixed effects linear models analyzed in GraphPad Prism, a 2way ANOVA on a dataset consolidated by reducing a non-significant effect and a Mixed effects linear model with explicitly defined interaction effects were subsequently explored to better understand the significance of the effects. For (2) linear models fitted in R, linear regression and residuals were plotted to determine if the linear model chosen was appropriate.

For linear models fitted, Fisher's least significant difference post hoc tests were computed either in GraphPad Prism or in R, and effect sizes of all statistical tests were estimated using $\widehat{\omega}_p^2$ calculated in R using the formula below:

$$\widehat{\omega}_p^2 = \frac{SS_{effect} - \left(df_{effect} \times MS_{error}\right)}{SS_{effect} + \left(N - df_{effect}\right) \times MS_{error}}$$

To detect outliers, robust Z-score of the median absolute deviation for a univariate data set X_1, X_2, ..., X_n was calculated using the formula and criterion below:

$$Z_{robust,i} = \frac{0.6745 \times \left(X_i - median(X)\right)}{median\ absolute\ deviation(X_i)}$$

$$where\ median\ absolute\ deviation(X_i) = median(|X_i - median(X)|)$$

$$and\ X_i\ is\ an\ outlier\ if\ \left|Z_{robust,i}\right| > 3.5$$

REFERENCES

Endo T, Maekawa F, Võikar V, Haijima A, Uemura Y, Zhang Y, Miyazaki W, Suyama S, Shimazaki K, Wolfer DP, Yada T, Tohyama C, Lipp HP, Kakeyama M. Automated test of behavioral flexibility in mice using a behavioral sequencing task in IntelliCage. Behav Brain Res. 2011 Aug 1;221(1):172-81. doi: 10.1016/j.bbr.2011.02.037.

Gramfort A, Luessi M, Larson E, Engemann DA, Strohmeier D, Brodbeck C, Goj R, Jas M, Brooks T, Parkkonen L, Hämäläinen M. MEG and EEG data analysis with MNE-Python. Front Neurosci. 2013 Dec 26;7:267. doi: 10.3389/fnins.2013.00267.

Pedregosa F, Varoquaux G, Gramfort A, Michel V, Thirion B, Grisel O, Blondel M, Prettenhofer P, Weiss R, Dubourg V, Vanderplas J, Passos A, Cournapeau D, Brucher M, Perrot M, Duchesnay É. Scikit-learn: Machine learning in Python. Journal of Machine Learning Research. 2011 Oct:12:2825–2830.

van der Walt S, Schönberger JL, Nunez-Iglesias J, Boulogne F, Warner JD, Yager N, Gouillart E, Yu T; scikit-image contributors. Scikit-image: image processing in Python. PeerJ. 2014 Jun 19;2:e453. doi: 10.7717/peerj.453.

Vinck M, Oostenveld R, van Wingerden M, Battaglia F, Pennartz CM. An improved index of phase-synchronization for electrophysiological data in the presence of volume-conduction, noise and sample-size bias. Neuroimage. 2011 Apr 15;55(4):1548-65. doi: 10.1016/j.neuroimage.2011.01.055.

Virtanen P, Gommers R, Oliphant TE, Haberland M, Reddy T, Cournapeau D, Burovski E, Peterson P, Weckesser W, Bright J, van der Walt SJ, Brett M, Wilson J, Millman KJ, Mayorov N, Nelson ARJ, Jones E, Kern R, Larson E, Carey CJ, Polat İ, Feng Y, Moore EW, VanderPlas J, Laxalde D, Perktold J, Cimrman R, Henriksen I, Quintero EA, Harris CR, Archibald AM, Ribeiro AH, Pedregosa F, van Mulbregt P; SciPy 1.0 Contributors. SciPy 1.0: fundamental algorithms for scientific computing in Python. Nat Methods. 2020 Mar;17(3):261-272. doi: 10.1038/s41592-019-0686-2.

Yoshinaga K, Matsuhashi M, Mima T, Fukuyama H, Takahashi R, Hanakawa T, Ikeda A. Comparison of Phase Synchronization Measures for Identifying Stimulus-Induced Functional Connectivity in Human Magnetoencephalographic and Simulated Data. Front Neurosci. 2020 Jun 19;14:648. doi: 10.3389/fnins.2020.00648.
