# Supplementary material for: GluK1 kainate receptors in parvalbumin interneurons modulate cortico-hippocampal network dynamics during social behavior
Source: Transl Psychiatry. 2026 Apr 30;16:313. doi: 10.1038/s41398-026-04060-z (PMC13276187; doi:10.1038/s41398-026-04060-z)
Supplement: Supplementary file 2 — Supplementary Figures and Tables [file 41398_2026_4060_MOESM2_ESM.pdf]

Figure S1

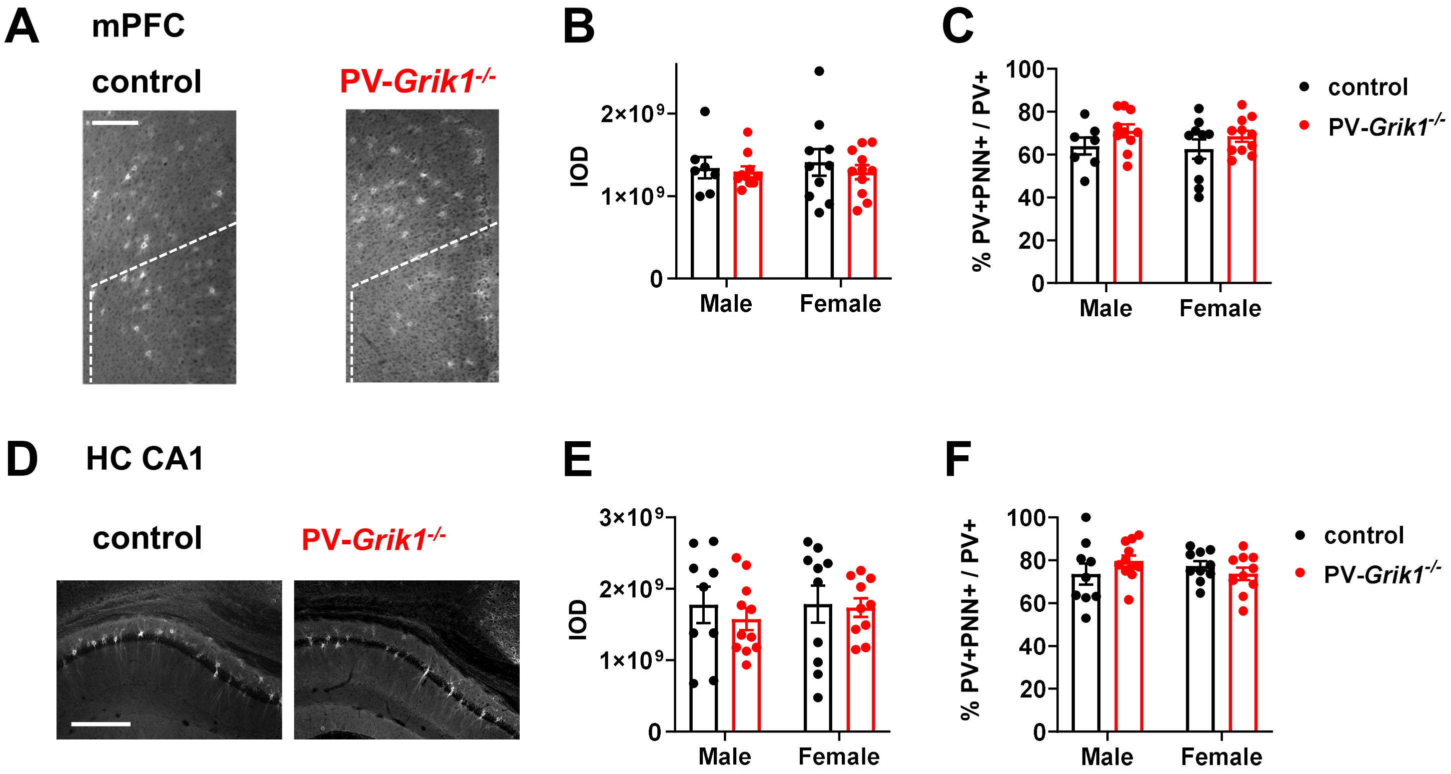

Figure S1. Absence of *Grik1* in PV INs has little to no effect on the perineuronal nets (PNNs)

A Representative images of Wisteria floribunda lectin (WFA) staining, representing PNN, in the mPFC of the male control and PV-*Grik1*$^{-/-}$ mice. Scale bar = 200 µm.

B Comparison of integrated optical density (IOD) of PNN labeling in PV INs across genotypes and sexes in the mPFC. Effect of genotype, $F_{(1, 34)}$=0.5058, p=0.4818, 2-way ANOVA.

C Comparison of proportion of PV INs surrounded by PNNs across genotypes and sexes. Effect of genotype, $F_{(1, 34)}$=3.424, p=0.0730, 2-way ANOVA.

D-F Corresponding data for HC CA1. Scale bar = 500 µm. Effect of genotype on PNN IOD, $F_{(1, 36)}$=0.3811, p=0.5409, 2-way ANOVA. Effect of genotype on % of PV IN that are PNN+, $F_{(1, 36)}$=0.1367, p=0.7137, 2-way ANOVA.

Figure S2

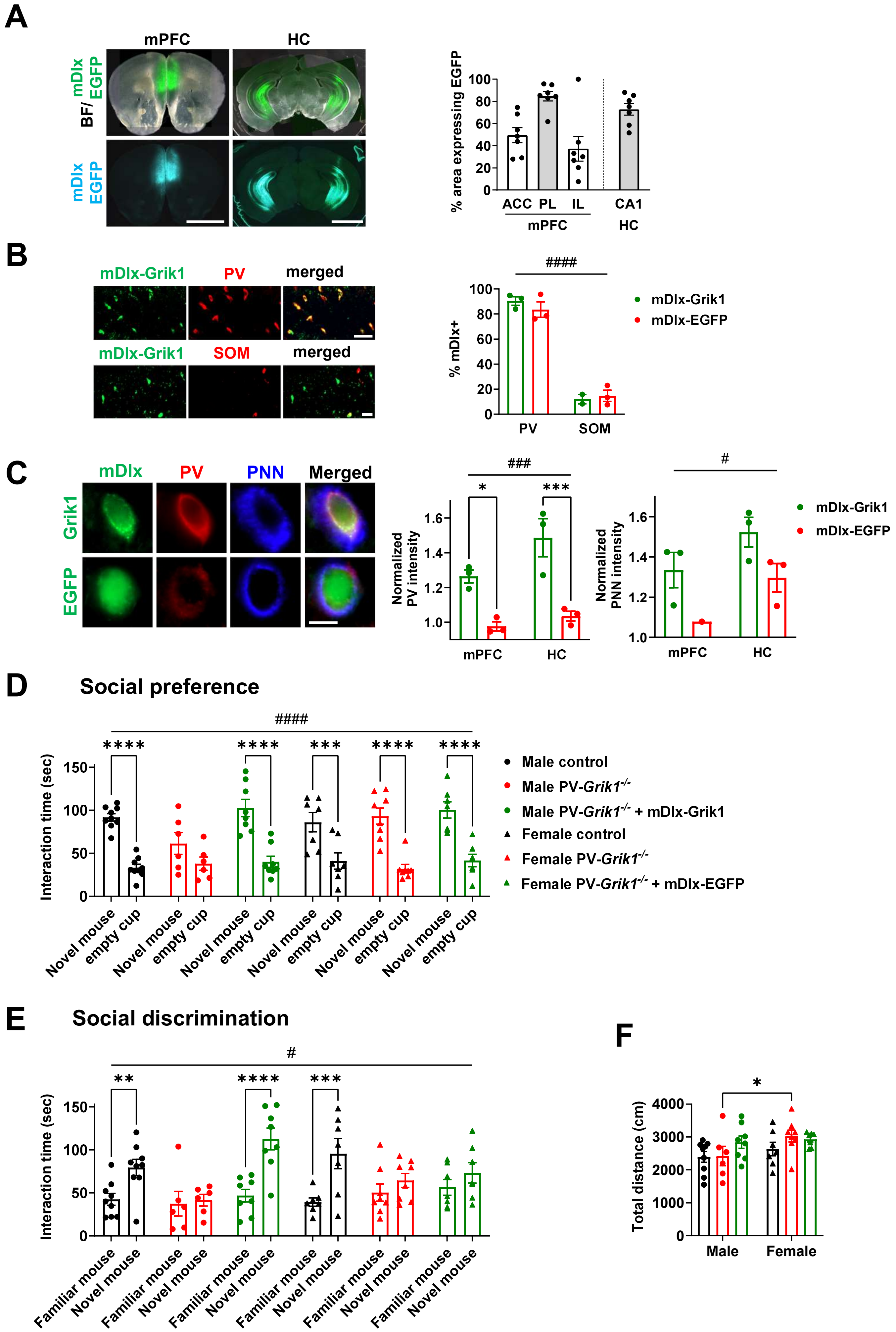
A
mPFC
HC
BF/ mDlx EGFP
mDlx EGFP
% area expressing EGFP
100
80
60
40
20
0
ACC PL IL
CA1
mPFC
HC
B
mDlx-Grik1
PV
merged
mDlx-Grik1
SOM
merged
####
% mDlx+
100
80
60
40
20
0
PV
SOM
mDlx-Grik1
mDlx-EGFP
C
mDlx
PV
PNN
Merged
Grik1
EGFP
###
*
***
Normalized PV intensity
1.6
1.4
1.2
1.0
mPFC
HC
#
Normalized PNN intensity
1.6
1.4
1.2
1.0
mPFC
HC
mDlx-Grik1
mDlx-EGFP
D
Social preference
####
****
****
***
****
****
Interaction time (sec)
150
100
50
0
Novel mouse
empty cup
Male control
Male PV-Grik1-/-
Male PV-Grik1-/- + mDlx-Grik1
Female control
Female PV-Grik1-/-
Female PV-Grik1-/- + mDlx-EGFP
E
Social discrimination
#
**
****
***
Interaction time (sec)
150
100
50
0
Familiar mouse
Novel mouse
F
*
Total distance (cm)
4000
3000
2000
1000
0
Male
Female

Figure S2. mDlx-driven expression of *Grik1* in the HC and mPFC rescues defects in PV expression and social behaviors in male PV-*Grik1*$^{-/-}$ mice.

A Representative images of EGFP expression in the mPFC and in the HC. % area expressing mDlx-EGFP in anterior cingulate cortex (ACC), prelimbic cortex (PL), and infralimbic cortex (IL). Data from mDlx-EGFP injected brains, n=7. Scale bar = 5 mm.

B Characterization of the cell types targeted by mDlx enhancer driven expression. Representative images of HC CA1 PV+, SOM+ (red) and mDlx-*Grik1* expressing (green) cells. Quantified data on the % HC CA1 EGFP+ cells that are PV+ or SOM+. Effect of cell type, $F_{(1,7)}$=227.6, $^{\#\#\#\#}$p<0.0001; effect of *Grik1*, $F_{(1,7)}$=0.1983, p=0.6695, 2-way ANOVA. Data pooled from 3-12 sections/mouse, 3 mice per group. Scale bar = 40 µm.

C Representative images and pooled data on the normalized mean intensity of PV (red) and PNN (blue) immunostaining in mDlx-*Grik1* and mDlx-EGFP expressing cells. Effect of *Grik1* on PV intensity, $F_{(1,\ 8)}$=36.90, $^{\#\#\#}$p=0.0003, ***p=0.0008, *p=0.0101, Fisher's least significant difference post hoc test. Effect of *Grik1* on PNN intensity, $F_{(1,\ 6)}$=6.451, $^{\#}$p=0.0441. Data pooled from 2-16 cells/slice, 3-4 sections/mouse, 3 mice per group. Scale bar = 10 µm.

D Analysis of the time spent interacting with a novel mouse vs empty cup, including the data from Figure 1C for control and PV-*Grik1*$^{-/-}$ mice as well as the additional data for male PV-*Grik1*$^{-/-}$ injected with AAV-mDlx-*Grik1* and female PV-*Grik1*$^{-/-}$ injected with AAV-mDlx-EGFP in the mPFC and HC. Stimulus mouse effect, $F_{(1,78)}$=119.6, p<0.0001, $\widehat{\omega}_p^2$=0.56, 2-way ANOVA. ****p<0.0001, ***p=0.0003, Fisher's least significant difference post hoc test. Male PV-*Grik1*$^{-/-}$ + mDlx-*Grik1* n=8, female PV-*Grik1*$^{-/-}$ + mDlx-EGFP n=7.

E Analysis of the time spent interacting with a novel vs familiar mouse, including the data from Figure 1D for control and PV-*Grik1*$^{-/-}$ mice, as well as the additional data for male PV-*Grik1*$^{-/-}$ injected with AAV-mDlx-*Grik1* and female PV-*Grik1*$^{-/-}$ injected with AAV-mDlx-EGFP in the the mPFC and HC. Interaction, $F_{(5,78)}$=2.788, p=0.0227, $\widehat{\omega}_p^2$=0.09, 2-way ANOVA. **p=0.0068, ***p=0.0004, ****p<0.0001, Fisher's least significant difference post hoc test. Male PV-*Grik1*$^{-/-}$ + mDlx-*Grik1* n=8, female PV-*Grik1*$^{-/-}$ + mDlx-EGFP n=7.

F Total distance travelled during the first 5 minutes of the habituation phase across genotypes and sexes, including the data from Figure 1F for control and PV-*Grik1*$^{-/-}$ mice. Effect of genotype, $F_{(2,39)}$=1.983, p=0.1513, 2-way ANOVA. *p=0.04, Fisher's least significant difference post hoc test. Male PV-*Grik1*$^{-/-}$ + mDlx-*Grik1* n=8, female PV-*Grik1*$^{-/-}$ + mDlx-EGFP n=7.

Figure S3

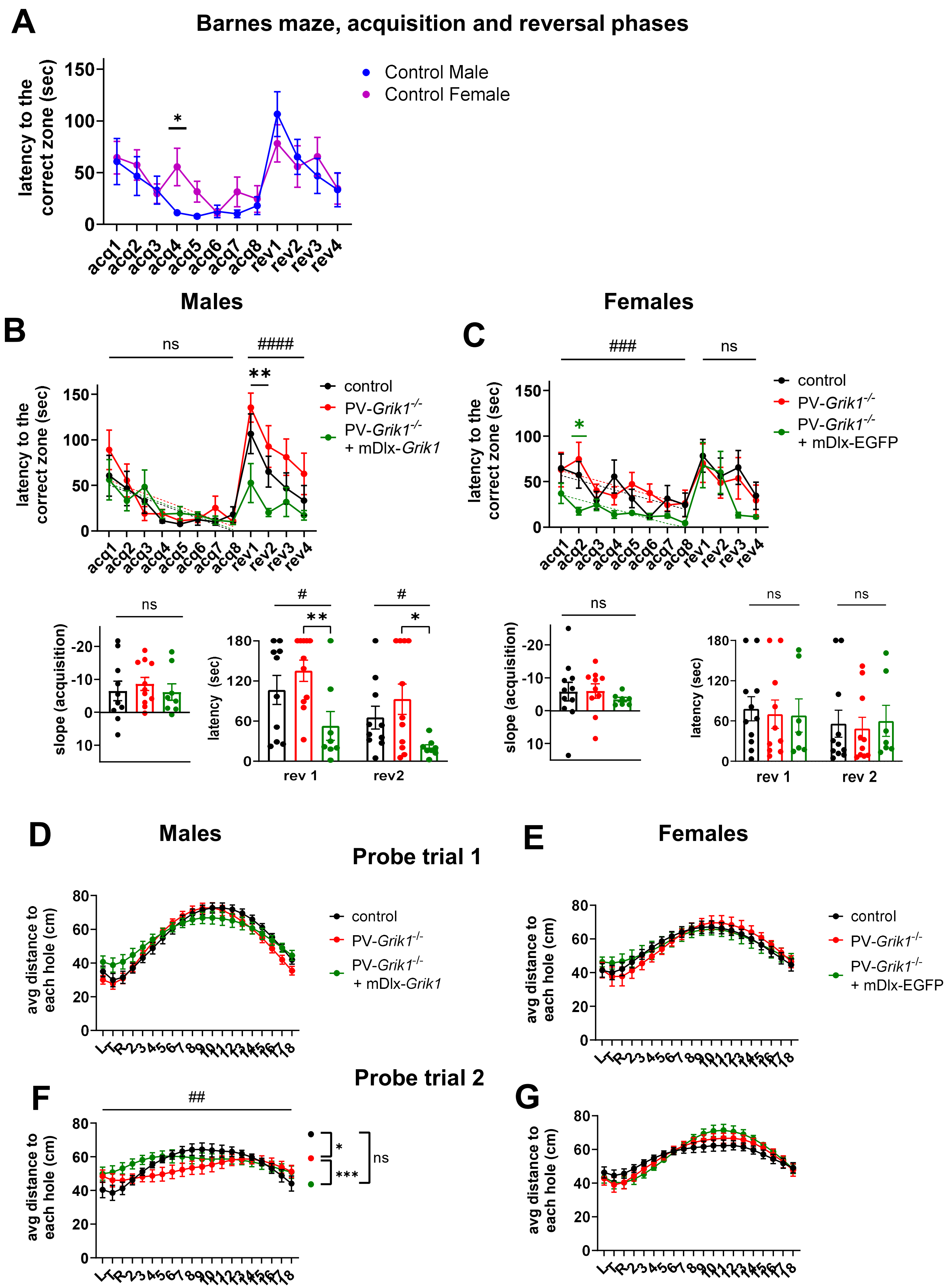
A
Barnes maze, acquisition and reversal phases
latency to the correct zone (sec)
Control Male
Control Female
acq1 acq2 acq3 acq4 acq5 acq6 acq7 acq8 rev1 rev2 rev3 rev4
Males
Females
B
C
ns
####
###
**
*
control
PV-Grik1-/-
PV-Grik1-/- + mDlx-Grik1
PV-Grik1-/- + mDlx-EGFP
slope (acquisition)
latency (sec)
rev 1
rev2
rev 2
#
D
E
Probe trial 1
avg distance to each hole (cm)
Probe trial 2
F
G
##
***
ns

Figure S3. mDlx-driven expression of *Grik1* in the HC and mPFC rescues defects in spatial re-learning.

A Comparison of trial-by-trial latencies to enter the correct zone with the escape box in the Barnes maze test, for control males and females, during eight acquisition trials (acq1-8) and four reversal acquisition trials (rev1-4). Effect of sex, $F_{(1,228)}$=1.502, p=0.2216, $\widehat{\omega}_p^2$=0.002, 2-way ANOVA. *p<0.05, Fisher's least significant difference post hoc test. Male n=10, female n=11. Dots represent mean ± SEM.

B Comparison of acquisition and reversal acquisition trials in the Barnes maze for males, including data from Figure 2A for control and PV-*Grik1*$^{-/-}$ mice, and the additional data for PV-*Grik1*$^{-/-}$ expressing mDlx-*Grik1* in the mPFC and HC**.** The Bar graphs show the slope of the learning curve during acq1-8 and the latencies during rev1 and rev2 for individual animals. Rev 1: effect of genotype, $F_{(2,26)}$=4.321, p=0.024, 1-way ANOVA. **p=0.007, Fisher's least significant difference post hoc test. Rev 2: effect of genotype, $F_{(2,26)}$ = 3.769, p=0.0365, 1-way ANOVA. *p=0.0109, Fisher's least significant difference post hoc test. Male PV-*Grik1*$^{-/-}$ + mDlx-*Grik1* n=8.

C Comparison of acquisition and reversal acquisition trials in the Barnes maze for females, including data from Figure 2B for control and PV-*Grik1*$^{-/-}$ mice, and the additional data for PV-*Grik1*$^{-/-}$ expressing mDlx-EGFP in the mPFC and HC. The bar graphs are shown as in B. Female PV-*Grik1*$^{-/-}$ + mDlx-EGFP n=7.

D Comparison of average distance from each hole during the 1st probe trial, for males. Effect of genotype, $F_{(2,520)}$=2.066, p=0.1277, mixed effects model.

E Similar data as in D, for females. Effect of genotype, $F_{(2,500)}$=0.0644, p= 0.9376, mixed effects model.

F Comparison of average distance from each hole during the 2nd probe trial, for males. Effect of genotype, $F_{(2,520)}$=6.676, $^{\#\#}$p= 0.0014, mixed effects model. Pairwise comparisons: control vs. PV-*Grik1*$^{-/-}$, $F_{(1,380)}$=4.811, *p=0.0289, 2-way ANOVA; control vs. PV-*Grik1*$^{-/-}$ + mDlx-*Grik1*, $F_{(1,320)}$=2.301, p=0.1303; PV-*Grik1*$^{-/-}$ vs. PV-*Grik1*$^{-/-}$ + mDlx-*Grik1*, $F_{(1,340)}$=12.18, ***p=0.0005, 2-way ANOVA.

G Similar data as in F, for females. Effect of genotype and AAV, $F_{(2,500)}$=1.386, p=0.2509, mixed effects model.

Figure S4

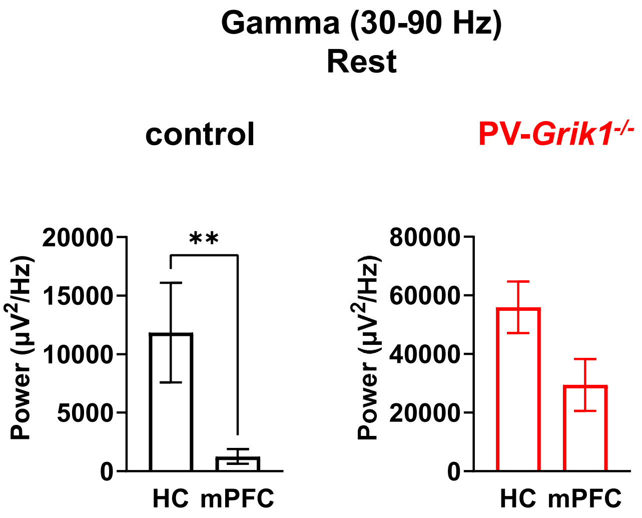

Figure S4. Brain region dependent differences in gamma power are attenuated in PV-*Grik1*$^{-/-}$ mice.

Comparison of gamma power between HC and mPFC. Control mice at rest (n=8): **p=0.0078, Wilcoxon matched-pairs signed rank test. PV-*Grik1*$^{-/-}$ mice at rest (n=9): p=0.078, paired t-test.

Table S5

	Fig 3B, % time interacting values			
	control littermate	control stranger	PV-*Grik1*$^{-/-}$ littermate	PV-*Grik1*$^{-/-}$ stranger
Z_{robust} $(Outlier\ if\ \lvert Z_{robust}\rvert > 3.5)$	2.81	2.81	2.29	2.29
	2.51	2.81	2.29	2.29
	1.91	2.81	2.29	2.23
	1.78	2.81	2.29	2.14
	1.14	2.81	2.29	1.99
	0.36	2.81	2.29	1.76
	0.15	2.81	2.29	1.04
	-0.72	2.25	0.11	-1.31
			-0.21	-1.70

Table S6

H_0 = The sample values come from a population that follows a normal distribution	Fig 3B, % time interacting values				residuals
	control littermate	control stranger	PV-*Grik1*$^{-/-}$ littermate	PV-*Grik1*$^{-/-}$ stranger	
D'Agostino-Pearson (K2)	p=0.92	p=0.92	p=0.92	p=0.92	p=0.92
Anderson-Darling (A2*)	p=0.94	p=0.94	p=0.30	p=0.30	p=0.20
Shapiro-Wilk (W)	p=0.97	p=0.97	p=0.36	p=0.36	p=0.27
Kolmogorov-Smirnov (distance)	p>0.1	p>0.1	p>0.1	p>0.1	p=0.1
